# Economic evaluation of a lifestyle intervention for individuals with overweight or obesity suffering from chronic low back pain (the BO2WL trial): a protocol for a health economic analysis

**DOI:** 10.1136/bmjopen-2024-098272

**Published:** 2025-06-20

**Authors:** Alexander Philipp Schurz, Tom Deliens, Melanie Liechti, Matteo Vanroose, Ron Clijsen, Jo Nijs, Anneleen Malfliet, Wouter Van Bogaert, Peter Clarys, Heiner Baur, Jan Taeymans, Nathanael Lutz

**Affiliations:** 1 Department of Movement and Sport Sciences, Vrije Universiteit Brussel, Brussels, Belgium; 2 Department of Health Professions, Bern University of Applied Sciences, Bern, Switzerland; 3 University of Bern, Bern, Switzerland; 4 Department of Physiotherapy, Human Physiology and Anatomy, Vrije Universiteit Brussel, Brussels, Belgium; 5 Pain in Motion International Research Group, Brussels, Belgium; 6 Pain in Motion Research Group (PAIN), Department of Physiotherapy, Human Physiology and Anatomy, Faculty of Physical Education and Physiotherapy, Vrije Universiteit Brussel, Brussels, Belgium; 7 Rehabilitation and Exercise Science Laboratory RESLab Department of Business Economics, Health, and Social Care, University of Applied Sciences and Arts of Southern Switzerland, Landquart/Manno, Switzerland; 8 International University of Applied Sciences THIM, Landquart, Switzerland; 9 Chronic pain rehabilitation, Department of Physical Medicine and Physiotherapy, University Hospital Brussels, Brussels, Belgium; 10 Department of Health and Rehabilitation, Unit of Physiotherapy, Institute of Neuroscience and Physiology, Sahlgrenska Academy, University of Gothenburg, Gothenburg, Västra Götaland County, Sweden; 11 Research Foundation Flanders, Brussels, Brussels, Belgium; 12 Faculty of Human Sciences, Institute of Sport Science, University of Bern, Bern, BE, Switzerland

**Keywords:** Back pain, Overweight, Obesity, HEALTH ECONOMICS, Chronic Pain

## Abstract

**Introduction:**

Low back pain (LBP) is highly prevalent across the Western world, including Switzerland (CH) and Belgium (BE), with women experiencing higher disability rates than men. Chronic LBP (CLBP), persisting beyond 3 months, leads to diminished health-related quality of life and increased healthcare and societal costs. Evidence indicates a direct relationship between body mass index (BMI) and CLBP severity, with high BMI significantly driving LBP-related healthcare expenses. Current evidence supports combining pain neuroscience education (PNE) with cognition-targeted exercise therapy (CTET) to effectively manage CLBP.

An economic evaluation alongside a randomised controlled trial, entitled ‘Lifestyle Intervention in Overweight/Obese CLBP Patients: an International Multi-centre RCT’ (BO2WL), will be conducted in CH and BE. It aims to evaluate the addition of a lifestyle intervention to PNE and CTET compared with PNE and CTET alone for treating individuals with CLBP and overweight or obesity. This protocol outlines the details of the economic evaluation of this trial.

**Methods and analysis:**

The trial consists of a 14-week intervention followed by a 52-week post-intervention follow-up. Primary outcomes include direct and indirect costs, as well as quality-adjusted life years and pain intensity. Cost data will be converted into 2026 Belgium € (BE) and 2026 Swiss Francs (CHF) for CH, with a discount rate of 3.5%. Incremental cost-utility and cost-effectiveness ratios will be calculated from a societal perspective for each country.

**Ethics and dissemination:**

Ethics approval was obtained by the local ethics committees (Bern, CH: project ID 2022–02210; Brussels and Geel, BE: BUN 1432022000296). Results of the main trial and economic evaluation will be submitted for publication in a peer-reviewed journal.

**Trial registration number:**

NCT05811624.

**STRENGTHS AND LIMITATIONS OF THIS STUDY**

- Prospectively designed economic evaluation conducted alongside a clinical randomised controlled trial.
- Multicountry economic evaluation of a lifestyle intervention for individuals with overweight or obesity with chronic low back pain.
- Cost-effectiveness and cost-utility evaluation included to facilitate country-specific decision-making.
- Chronic low back pain with fluctuating pain values, complexity of underlying overweight and obesity, time horizon potentially not capturing long-term effects and differing cost data collection sources across Switzerland and Belgium.

## Introduction

 Low back pain (LBP) is reported as one of the most prevalent health issues across the Western world, including in Switzerland (CH) and Belgium (BE).^1^ Women in both countries exhibit higher rates of years lived with disability (YLD) compared with men (CH: 1343 YLDs per 100 000 (men) and 1827 YLDs per 100 000 (women); BE: 1132 YLDs per 100 000 (men) and 1570 YLDs per 100 000 (women)).^1^ A systematic review published in 2023 summarised the current evidence on direct and indirect costs of LBP in Europe, America and Western Pacific, estimating these costs to range from €2.3 billion to €2.6 billion and €0.24 billion to €8.15 billion for direct and indirect costs, respectively.^2^ In CH, health insurance costs between 2015 and 2019 for individuals with LBP were 72% higher compared with those without this condition, with presenteeism (ie, decreased work productivity due to health problems) identified as the primary cost driver.^3 4^ In BE, LBP is among the costliest musculoskeletal disorders, alongside neck pain and osteoarthritis, based on direct expenses and absenteeism (ie, work absence due to health problems) costs between 2015 and 2018.^5^ Moreover, in BE, a single episode of LBP leads to €3269 in direct and €6557 in indirect annual costs on average.^5^

When LBP in patients persists beyond 3 months, it is clinically classified as chronic LBP (CLBP).^6^ CLBP was found to correlate with diminished health-related quality of life, even higher healthcare expenses and higher societal costs compared with individuals without CLBP.^7^,^9^ Numerous studies have established a proportional relationship between body mass index (BMI) and the severity of pain, highlighting the association between higher BMI and CLBP.^10^,^15^ Chen, Fong and Wong evaluated the economic outcomes of musculoskeletal disorders attributable to high BMI in 192 countries in 2019.^16^ These authors found that BMI was a key driver for 36.3 million cases of LBP worldwide, accounting for healthcare costs of US$23.1 billion worldwide (2024 BE €22.4 billion).^16 17^

To address CLBP and overweight or obesity, proof-of-concept studies suggest a combined intervention of a behavioural weight management programme with a specific pain-focused treatment, including pain neuroscience education (PNE) and cognition-targeted exercise therapy (CTET).^1518^,^21^ The American College of Lifestyle Medicine recommended targeting both diet, including moderate dietary restriction (300– 500 kcal restriction per day), and physical activity levels for the treatment of overweight and obesity.^22^ In a recently published systematic review on the health economic evaluation of added weight loss interventions for people with musculoskeletal conditions and overweight or obesity, weak but consistent evidence towards the cost-effectiveness of these interventions was found.^23^ One Australian study included in this review evaluated in a trial-based economic evaluation a telephone-based lifestyle intervention specifically for individuals with CLBP.^24^ It was found out that the added telephone-based lifestyle intervention led to €267 lower healthcare costs, €829 lower absenteeism costs and thus cost-effectiveness of the lifestyle intervention from a societal perspective.^24^

This present trial builds and extends the current state-of-the-art by addressing both CLBP and overweight or obesity. It examines the (cost-) effectiveness of a healthy lifestyle intervention (ie, a behavioural weight reduction programme) combined with PNE and CTET and compares it with PNE and CTET alone for individuals with overweight or obesity and CLBP living in CH or BE. The focus of this health economic analysis plan is to describe the trial-based, embedded health economic evaluation of the planned trial.

The primary objective of the health economic evaluation is to investigate whether this lifestyle intervention in addition to PNE and CTET results in higher health-related quality of life, lower healthcare use and reduced societal costs compared with PNE and CTET alone in individuals with CLBP and overweight or obesity living in CH or BE.

## Methods and analysis

This present health economic protocol follows the ‘Content of Health Economics Analysis Plans (HEAPs) for Trial-Based Economic Evaluations’ reporting guidelines for trial-based economic evaluations.^25^

### Trial design

The study is an international, multicentre, randomised controlled trial being conducted in CH and BE entitled ‘Lifestyle Intervention in Overweight/Obese CLBP Patients: an International Multi-centre RCT’ (short title: Back Pain Overweight Obese Weight Loss Study, acronym: BO2WL). The study has been registered on clinicaltrials.gov (ID: NCT05811624) and was approved by the local ethics committees (Bern, CH: project ID 2022–02210; Brussels and Geel, BE: BUN 1432022000296). A total of 252 patients (126 per country) will be recruited for the trial, with the sample size calculated using the clinical parameter of mean pain intensity (as assessed by the Brief Pain Inventory) and not a cost outcome. Randomisation is stratified by weight category, sex and treatment location. Recruitment began in May 2023 and is scheduled to be completed in December 2025. This will be followed by a 12-month follow-up period extending until December 2026. At present (17 December 2024), 79 patients are included in the trial in CH and BE. Patients and the public were not involved in designing this trial.

### Study population

This study is being conducted in Bern (CH), Brussels and Geel (BE) and includes individuals aged between 18 years and 65 years, who have proficient verbal and written skills in the German (CH) or Dutch language (BE), respectively. Additionally, the participants must have chronic non-specific LBP for at least 3 months and be currently seeking care for this condition. Individuals who also experience leg pain related to the LBP rated below 7 out of 10 on a numeric rating scale (NRS) are also eligible for participation in this study. In addition, participants should have a BMI equal to or exceeding 25 kg/m^2^ to be considered overweight or equal to or exceeding 30 kg/m^2^ to be categorised as obese. Individuals with a BMI above 40 kg/m^2^ or patients with a healthy fat percentage, leg pain rating higher than 7 out of 10 on a NRS or with specific spinal pathologies (eg, spinal stenosis, hernia, spinal fracture or malignancy) will be excluded from the study. Furthermore, pregnant women, women who were pregnant in the past year or those in clarification or currently receiving invalidity payments will not be eligible to participate.

### Study interventions

Participants are blinded and randomly assigned to either the intervention group or the control group, with study assessors and statistician blinded to group allocation. All participants receive the same 14-week treatment depending on their group allocation. Participants in the intervention group will receive up to 17 individually based treatment sessions, supervised by experienced physiotherapists who have been trained a priori by the research team for these treatments, along with one home-based session. Treatment sessions will include PNE, CTET and a behavioural weight reduction programme. The latter is integrated alongside CTET therapy sessions with various nutrition-specific inputs and actions to increase physical activity. The participants of the control group also receive 17 individually based treatment sessions and one home-based session consisting of PNE and CTET therapy alone.

Physical therapists will document the content of each treatment session as required by their workplace. After treatment completion, the physical therapist will be asked to complete a questionnaire on therapy adherence and compliance of the participant (number of sessions, sessions per intervention, total duration, duration per intervention and overall compliance to the intervention (control or intervention group)).

### Economic approach

This planned health economic evaluation aims to assess whether the addition of a behavioural weight reduction intervention to PNE and CTET is cost-effective in comparison to PNE and CTET alone in the treatment of adult individuals with overweight or obesity and CLBP living in CH or BE. The main objective is to assess the cost-effectiveness and cost-utility from the start of treatment until 12 months after the end of the combined therapy, that is, within 66 weeks in total. Depending on the available data and literature, a model-based approach may be added after the complete collection of data from the BO2WL trial to extend the evaluated time horizon.

The trial-embedded economic analysis will be performed based on individual patient data from the BO2WL trial. Cost-effectiveness or cost-utility planes will be developed to present the incremental values of the costs and clinical effects (ie, pain) with 95% CIs. Additionally, if either the intervention or control group is not considered dominant, the incremental ratios for both the costs and clinical effects (ie, pain) or utilities will be calculated, being the incremental cost-effectiveness ratio and incremental cost-utility ratio, respectively, and plotted on the already mentioned planes. The evaluation will be conducted separately for each country, as there is currently no reliable method for economic evaluation in multinational trials.^26^ CH and BE are countries with different social healthcare systems, that is, per capita flat rates (CH) and income-related contributions (BE), respectively. The trial-based health economic analysis will be performed from a healthcare as well as from a societal perspective. The time horizon includes the 14-week intervention period and a 12-month follow-up period, totalling 66 weeks.

### Economic data collection and management

RStudio V.2024.04.1+748 or newer will be used for the statistical analysis.^27^ Cost-specific data will be collected using questionnaires at baseline (T0), after treatment completion (T1) and 3-month (T2), 6-month (T3), 9-month (T4) and 12-month (T5) follow-up. To evaluate direct medical costs (eg, health professional consultations, medication) and direct non-medical costs (eg, care provided by family and friends, travel to treatment locations), the German or Dutch version of the Medical Consumption Questionnaire, translated and adapted to each country’s specific needs, will be used (online supplemental files 1-3).^28^ Intervention costs due to study-related therapy will be assessed within the adherence report which is filled out by the physiotherapist after therapy completion. Indirect cost data (eg, costs due to productivity loss by absenteeism or presenteeism) will be collected using the Productivity Cost Questionnaire (iPCQ) (online supplemental files 1-3).^29 30^ Intangible costs will be excluded to prevent double counting, as there is some overlap between productivity and intangible costs.^31^,^33^ All questionnaires are accessed and filled out by the study participants online using the REDCap software (Vanderbilt University, Tennessee, USA).^34 35^ Table 1 includes a summary of the collected data.

**Table 1:** Summary of the economic evaluation framework.

Research question	Is the lifestyle intervention in addition to PNE and CTET compared with PNE and CTET alone cost-effective in the treatment of individuals with overweight or obesity suffering from chronic low back pain?		
Population	Individuals with chronic low back pain and overweight or obesity		
Intervention	PNE+ CTET + lifestyle intervention		
Comparator	PNE+ CTET		
Recruitment period	May 2023 to December 2025		
Time horizon	66 weeks		
Perspective	Healthcare sector and societal perspective		
Healthcare resource use	*Healthcare sector perspective*Intervention costsDirect medical costs (iMCQ)Direct non-medical costs	iMCQiMCQ	T0–T1T0, T1, T2, T3, T4, T5T0, T1, T2, T3, T4, T5
	*Societal perspective*Intervention costsDirect medical costsDirect non-medical costsIndirect costs	iMCQiMCQiPCQ	T0–T1T0, T1, T2, T3, T4, T5T0, T1, T2, T3, T4, T5T0, T1, T2, T3, T4, T5
Outcome measures	Primary outcome: quality of life	EuroQol 5 Dimensions - 5 Levels Questionnaire	T0, T1, T2, T3, T4, T5
	Secondary outcome: mean pain intensity	BPI	T0, T1, T2, T3, T5
Evaluation	Incremental costsIncremental effects/utilitiesCost-effectiveness/cost-utility planeICERs/ ICURs		T5–T0T5–T0T5–T0T5–T0
Currency	BE: Belgium € (2026)CH: Belgium € (2026), Swiss Francs (CHF, 2026)		
Discount rate	CH/BE: 3.5% costs and effects		
Sensitivity analyses	Probabilistic: variation of all input variables in nonparametric bootstrappingDeterministic: one-way sensitivity analysisScenario analysis: variation of discount rate (eg, country specific rates BE: 3.0% costs, 1.5% effects)		

BE, Belgium; BPI, Brief Pain Inventory; CH, Switzerland; CTET, cognition-targeted exercise therapy; ICER, Incremental Cost-Effectiveness Ratio; ICUR, Incremental Cost-Utility Ratio; iMCQ, Medical Consumption Questionnaire; iPCQ, Productivity Costs Questionnaire; PNE, pain neuroscience education; T0, baseline measurement; T1, post intervention, 14 weeks; T2, 3 months follow-up; T3, 6 months follow-up; T4, 9 months follow-up; T5, 12 months follow-up.

Costs will be gathered and presented in disaggregated form (ie, cost=units consumed × unit cost). The resource use will be valued in monetary terms using Swiss and Belgium unit costs at the respective assessment year (2023–2026).

Table 2 depicts the references used for the valuation of resource use.

**Table 2:** References for the valuation of resource use.

Cost category	Category	Switzerland	Belgium
Intervention costs	Treatment	17 Physiotherapy sessions: monetised according to current physiotherapy tariffs^61^,^63^1 Home-based session: not monetised, as the physiotherapist is not involved.Additional costs for lifestyle interventions: not collected	
Direct medical costs	GP	Tax points according to current Tariff^64^Value for one tax point Canton of Bern^65^	Valuation based on current fees^66^
	Specialists	Tax points according to current Tariff^64^Value for one tax point Canton of Bern^65^	Valuation based on current fees^66^
	Company doctor	135 CHF per consultationMedical consultant (eg, insurances): 300–500 CHF/ consultation	According to median salary, paid by employer^67^
	Physical therapist	Tax points according to current Tariff^61^Value for one tax point Canton of Bern^62^	Valuation based on current fees^63^
	Occupational therapist	Tax points according to current Tariff^68^Value for one tax point Canton of Bern^69^	According to nomenclature 794732^70^
	Logopaedist	Tax points according to current Tariff^71^Value for one tax point Canton of Bern^72^	Valuation based on current fees^73^
	Nutritionist	Tax points according to current Tariff^74^Value for one tax point Canton of Bern^75^	According to nomenclature 771794^76^
	Psychologist, Psychotherapist, Psychiatrist	Tax points according to current Tariff^77 78^Value for one tax point^79^	Valuation based on current fees^80^
	Complementary medicine (eg, Homeopathy, Acupuncture)	If delivered by a medical doctor, according to valuation of GP consultations.If not delivered by a medical doctor, the mean price of three practices for complementary medicine will be used for the valuation.	Based on current tariffs^81 82^
	Social worker	Calculation based on cantonal salary system (salary class 15–19)^83^	Valuation based on the median salary, paid by employer^76^
	Medication	Specialities list^84^If not included: mean of medication price will be calculated from three web-based pharmacies.	Valuation based on current fees^85^
	Hospital stays, day-care treatments	According to current SwissDRG System^86^SwissDRG-Baserate^87^	Valuation based on current fees^66^
	Ambulance	According to currently reported rates^88^	According to reported rates^89^
	Outpatient clinic	Like specialists’ consultation	Like specialists’ consultation
Direct non-medical costs	Domestic care	According to Standard employment contract for employees in housekeeping (NAV Housekeeping, Article 5)^90^	Valuation based on current fees^91^
	Personal, nursing care	According to Ordinance of the Federal Department of Home Affairs on benefits in the compulsory healthcare insurance (KLV, Article 7 a)^92^	Valuation based on current fees^91^
Indirect costs	Absenteeism, presenteeism	(iPCQ-question 8)* (iPCQ-question 9/10) * daily wage Switzerland	(iPCQ-question 8)* (iPCQ-question 9/10) * daily wage Belgium
	Hourly wage	Annual income before tax^93^Paid vacation^94^Public holidays^94^Hours standard workday^95^	Annual income before tax^93^Paid vacation^96^Public holidays^97^Hours standard workday^98^

CHF, Swiss Francs; GP, General practitioner; iPCQ, Productivity Costs Questionnaire; KLV, Krankenpflege-Leistungsverordnung - Nursing service regulation; NAV, Normalarbeitsvertrag für Arbeitnehmerinnen und Arbeitnehmer in der Hauswirtschaft - Standard employment contract for employees in housekeeping.

Belgium related data will be reported in 2026 Belgium € and Swiss data in both 2026 Belgium € and 2026 CHF. Costs will be adjusted for inflation using the gross domestic product (GDP). Swiss related data will be converted into the respective currency using purchasing power parities (PPP) for GDP. This calculation will be done using a web-based tool which includes the following formula^36^:

Cost*_2023 Euro_ =* $\frac{{GDP}_{2}*{PPP}_{2}}{{GDP}_{1}*{PPP}_{1}}$* Cost*_original_*

with GDP_1_ implying the GDP deflator index for the study currency in the referenced price year; GDP_2_ referring to the GDP deflator index for the study currency in the price year 2026; PPP_1_ being the PPP conversion rate for the study currency in the price year 2026; PPP_2_ being the PPP conversion rate for the target currency^37^ in the price year 2026 and finally cost_original_ being the price in the study currency in the original currency.^36^

The primary outcome measure of the health economic evaluation will be quality-adjusted life years (QALYs) derived from utility scores, obtained using the EuroQol - 5 Dimensions - 5 Levels (EQ-5D-5L) quality of life instrument at each time point (T0–T5) (online supplemental files 1-3).^38^ Utility scores will be calculated from the EQ-5D-5L values and using utility values for Germany, England and Belgium using the NICE (National Institute for Health and Care Excellence)-recommended approach, employing a mapping function from EQ-5D-5L.^39^,^41^ The clinical outcome that will be used in the cost-effectiveness acceptability will be the primary outcome of the trial, which is the evaluation of the average pain using the Brief Pain Inventory questionnaire.^42^,^44^ Pain will be rated using a numeric analogue scale ranging from 0 to 10 (higher scores indicating more severe pain, T0, T5).

Baseline (T0), post-intervention (T1) and 12-month follow-up measurements (T5) will take place at the respective university assessment location (Bern Movement Laboratory of Bern University of Applied Science, CH; Vrije Universiteit Brussel and Sint-Dimpna hospital, Geel, BE).

### Economic data analysis

The full analysis set comprises all participants randomised, aligning with the ‘intention-to-treat’ principle. The per-protocol set comprises individuals from the full analysis set without any significant protocol violations. The primary analysis will occur after the assessment of all participants at the 12-month follow-up time point (T5). Discounting of costs and outcomes over a time horizon exceeding 12 months will follow NICE guidelines, with a discount rate of 3.5% applied to both costs and outcomes in CH and BE. Country-specific discount rates for BE will be applied in the scenario analysis using 3.0% for costs and 1.5% for outcomes.^45^ Mean differences in costs and utilities or pain intensity of the treatment groups will be calculated, accompanied by their 95% CIs. Indirect costs will include costs via absenteeism and presenteeism. The human capital approach will be applied to calculate the indirect costs by multiplying the hours of absence or reduced productivity with the average country-specific salary. While presenteeism and absenteeism time are evaluated through the iPCQ questionnaire at each time point, the hourly wage is estimated based on the average annual income in each country.^3646^,^50^ Therefore, the following formula is used to calculate the hourly wage as previously used in another study^51^:

Hourly wage =

$\frac{Annual\;income\;before\;tax}{52\;x\;5\;–\;\left(Paid\;vacation\;days\;+\;Public\;holidays\right)}/\;Hours\;of\;standard\;workday.$

Mean differences in resource use, costs, QALYs and pain values between the two groups will be calculated with associated 95% CIs separately per country (CH, BE).

The comparison of mean costs between the groups will be initially conducted using Ordinary Least Squares^52^ regression, adjusting for the minimisation variables used in the randomisation procedure. Subsequently, the residuals from the ordinary least-squares (OLS) regression model will be examined to assess their distribution. Based on this assessment, it will be determined whether OLS is appropriate or if an alternative regression model, such as generalised linear models, should be considered. To analyse the outcome variables, an appropriate regression model will also be employed to account for any imbalances in baseline utility and the minimisation variables from the randomisation process.^53^

Face validity tests will always be conducted by the same study assessors on all data on completion of the respective questionnaires in the REDCap software.^53 54^ In case of misspelling or unclear information in the respective questionnaires, the participant is contacted directly via email or telephone all the time by the same study assessors. Corrections will be documented at the respective question on REDCap directly. Trial data will be reviewed for any missing entries. If missing data are identified close to the respective assessment time point, the participant will be contacted by the same set of study assessors through REDCap, email or phone to complete the necessary questionnaire or section. Should it prove impossible to retrieve the missing data, the chosen method will depend on the extent of the missing data and the reasons for its absence. For data missing at random, multiple imputation methods may be used.^53 55^ In case of non-random missing data, parametric bootstrapping will be used.

The assessed overall mean incremental costs, effectiveness measures and utilities from T0 to T5 comparing each treatment group will be graphically represented in a cost-effectiveness or cost-utility plane. While the healthcare sector perspective considers intervention costs, direct medical and non-medical costs, the societal perspective adds the indirect costs to the cost evaluation.^56 57^ Additionally, if a group is not already considered dominant in the cost-effectiveness/utility plane, incremental cost-effectiveness (ICER) and cost-utility ratios (ICUR) will be calculated using the following formula:

ICER or ICUR = $\frac{{Costs}_{Interventiongroup}-{Costs}_{Controlgroup}}{{Effects}_{Interventiongroup}-{Effects}_{Controlgroup}}$

The two involved countries (CH and BE) do not have specific willingness-to-pay thresholds that determine decision making.^58^ Therefore, this will also be adopted in the analysis, and willingness to pay will be varied in the probabilistic sensitivity analysis to increase decision making. A non-parametric bootstrapping method will be employed to assess the sampling uncertainty associated with the mean ICER and ICUR values. This procedure will generate 10 000 estimates of incremental costs and effects^59^ and graphically present them in a cost-effectiveness acceptability curve.

Subgroup analyses will be performed using the complete cases to explore variations in cost-effectiveness and cost-utility across CH and BE and distinct patient subgroups (eg, different weight categories). In the scenario analysis, varying discount rates (eg, country-specific discount rates for BE with 3.0% for costs and 1.5% for outcomes) and different value sets for the EQ-5D-5L questionnaire will be applied to assess their effect on the outcome of cost-effectiveness.

### Reporting and publishing

The reporting of the final health economic evaluations in peer-reviewed journals will adhere to the Consolidated Health Economic Evaluation Reporting Standards 2022 guideline.^60^ Any discrepancies from the predetermined HEAP will be described and rationalised within the final published report.

## Supplementary material

10.1136/bmjopen-2024-098272 online supplemental file 1

10.1136/bmjopen-2024-098272 online supplemental file 2

10.1136/bmjopen-2024-098272 online supplemental file 3

## Data Availability

No data are available.
