# Supplementary material for: Economic evaluation of a lifestyle intervention for individuals with overweight or obesity suffering from chronic low back pain (the BO2WL trial): a protocol for a health economic analysis
Source: BMJ Open. 2025 Jun 20;15(6):e098272. doi: 10.1136/bmjopen-2024-098272 (PMC12184353; doi:10.1136/bmjopen-2024-098272)
Supplement: online supplemental file 1 [file bmjopen-15-6-s001.pdf]

# Vragenlijst uw gezondheid en werk

**De vragenlijst gaat over uw gezondheid en werk in de afgelopen 3 maanden. We beginnen met enkele algemene vragen zoals uw geslacht en geboortedatum.**

**Het duurt ongeveer 10 minuten om de lijst in te vullen.**

Hebt u betaald werk?

◯ Nee
◯ Ja

De volgende vragen gaan over uw baan. Dus over werk waarvoor u betaald wordt.

Wat is uw beroep?

__________________________________
(uw beroep)

Hoeveel uur per week werkt u? Tel alleen de uren waarvoor u betaald wordt.

__________________________________
(uren)

Op hoeveel dagen in de week werkt u?

__________________________________
(op ... dagen)

Bent u in de afgelopen 3 maanden afwezig geweest van uw werk omdat u ziek was?
((Tel alleen de werkdagen in de afgelopen 3 maanden))

◯ Nee
◯ Ja, ik ben verschillende dagen afwezig geweest. Namelijk {ipcq_be_q4_days} dagen.

Was u langer dan de gehele periode van 3 maanden afwezig van uw werk doordat u ziek was?

Het gaat om een aaneengesloten periode van werkverzuim.

◯ Nee
◯ Ja

Wanneer heeft u zich ziek gemeld?

__________________________________

Waren er in de afgelopen 3 maanden dagen waarop u wel gewerkt heeft, maar tijdens uw werk last had van lichamelijke of psychische problemen?

◯ Nee
◯ Ja

---

Op hoeveel werkdagen had u tijdens uw werk last van uw lichamelijke of psychische problemen?

Tel alleen de werkdagen in de afgelopen 3 maanden.

______________________________
(aantal werkdagen)

---

Op de dagen dat u last had, kon u misschien niet zoveel werk doen als normaal. Hoeveel werk kon u op deze dagen gemiddeld doen? Kijk naar de cijfers hieronder. Een 10 betekent dat u op deze dagen net zoveel kon doen als normaal. Een 0 betekent dat u op deze dagen niets kon doen.

- 0 = Ik kon op deze dagen niets doen
- 5 = Ik kon ongeveer de helft doen
- 10 = Ik kon net zoveel doen als normaal

0 5 10

*(Place a mark on the scale above)*

**Ook bij onbetaald werk, kunt u last hebben van uw lichamelijke of psychische problemen. Soms kunt u daardoor minder doen. U kunt bijvoorbeeld niet goed voor de kinderen zorgen of vrijwilligerswerk doen. Of geen boodschappen doen of in de tuin werken. Daarover gaan de volgende vragen.**

Waren er dagen waarop u minder onbetaald werk kon doen door uw lichamelijke of psychische problemen? Het gaat om dagen in de afgelopen 3 maanden

◯ Nee
◯ Ja

---

Op hoeveel dagen was dit zo? Tel alleen de dagen in de afgelopen 3 maanden.

__________________________________

---

Stel dat iemand, bijvoorbeeld uw partner, familielid of een bekende, u op deze dagen had geholpen. En al het onbetaalde werk wat u niet kon doen, voor u had gedaan. Hoeveel uur was die persoon hier op deze dagen dan gemiddeld mee bezig geweest?

__________________________________
(gemiddeld uur op deze dagen)

---

Dit was de laatste vraag van deze vragenlijst.

Misschien heeft u nog vragen of opmerkingen? Schrijft u deze dan hieronder op.

Hartelijk dank!

__________________________________

# Vragenlijst zorggebruik

**De vragenlijst gaat over uw zorggebruik in de afgelopen 3 maanden. We beginnen met algemene vragen. Daarna stellen we vragen over uw zorggebruik.**

**Het duurt ongeveer 20 minuten om de lijst in te vullen.**

**Welke afspraken tellen mee?**
**- Controles**
**- Afspraken omdat u een lichamelijke of psychische klacht had**
**- Afspraken waarbij de dokter bij u thuis kwam**
**- Telefonische afspraken**
**- Telefoontjes met de receptenlijn**

**Wat telt niet mee?**
**Afspraken voor een ander, bijvoorbeeld voor uw kind.**
**Telefoontjes om een afspraak te maken.**
**Weet u niet precies hoeveel afspraken u had, schrijf dan op hoeveel het er ongeveer waren.**

Vragen over zorggebruik

Heeft u in de afgelopen3 maanden een gezondheidswerker geraadpleegd? (bijvoorbeeld: huisarts, verpleegkundige, maatschappelijk werker, kinesitherapeut, ergotherapeut, logopedist, diëtist, homeopaat/acupuncturist, psycholoog/psychiater/psychotherapeut of bedrijfsarts)

◯ Nee
◯ Ja

Heeft u in de afgelopen 3 maanden een afspraken gehad met een huisarts?

◯ Nee
◯ Ja

Hoeveel afspraken had u in de afgelopen 3 maanden met uw huisarts?

__________________________________

Hoeveel afspraken had u in de afgelopen 3 maanden met een maatschappelijk werker?

◯ Geen enkele afspraak
◯ ... {imcq_be_q2a} afspraken

Hoeveel afspraken had u in de afgelopen 3 maanden met een kinesitherapeut?

◯ Geen enkele afspraak
◯ ... {imcq_be_q3a} afspraken

Hoeveel afspraken had u in de afgelopen 3 maanden met een ergotherapeut?

◯ Geen enkele afspraak
◯ ... {imcq_be_q4a} afspraken

Hoeveel afspraken had u in de afgelopen 3 maanden met een logopedist?

◯ Geen enkele afspraak
◯ ... {imcq_be_q5a} afspraken

Hoeveel afspraken had u in de afgelopen 3 maanden met een diëtist?

◯ Geen enkele afspraak
◯ ... {imcq_be_q6a} afspraken

Hoeveel afspraken had u in de afgelopen 3 maanden met een homeopaat? Of met een acupuncturist? Tel alle afspraken met deze zorgverleners bij elkaar op.

◯ Geen enkele afspraak
◯ ... {imcq_be_q7a} afspraken

Hoeveel afspraken had u in de afgelopen 3 maanden met een psycholoog? Of met een psychotherapeut of psychiater? Tel alle afspraken met deze zorgverleners bij elkaar op.

◯ Geen enkele afspraak
◯ ... {imcq_be_q8a} afspraken

Hoeveel afspraken had u in de afgelopen 3 maanden met de bedrijfsarts?

◯ Geen enkele afspraak
◯ ... {imcq_be_q9a} afspraken

## Zorgdiensten

Heeft u in de afgelopen 3 maanden hulp van de thuiszorg gehad?

Bijvoorbeeld:

- Huishoudelijke hulp (bv: stofzuigen, bed opmaken, boodschappen doen)
- Verzorging van uzelf (bv: hulp bij douchen of aankleden)
- Verpleging (bv: verband omdoen, medicijnen geven, bloeddruk meten)

◯ Nee
◯ Ja

---

Wat voor hulp van de thuiszorg heeft u gehad in de afgelopen 3 maanden?

☐ Huishoudelijke hulp (bv: stofzuigen, bed opmaken, boodschappen doen)
☐ Verzorging van uzelf (bv: hulp bij douchen of aankleden)
☐ Verpleging (bv: verband omdoen, medicijnen geven, bloeddruk meten)

---

Hoeveel weken heeft u deze thuiszorg gehad? Tel alle weken in de afgelopen 3 maanden bij elkaar op.

---

Aantal weken 'huishoudelijke hulp' (bv: voorbeeld: stofzuigen, bed opmaken, boodschappen doen) in de afgelopen 3 maanden.

______________________________
(In weken)

---

Aantal weken 'Verzorging van uzelf' (bv: hulp bij douchen of aankleden) in de afgelopen 3 maanden.

______________________________
(In weken)

---

Aantal weken 'Verpleging: (bv: verband omdoen, medicijnen geven, bloeddruk meten) in de afgelopen 3 maanden.

______________________________
(In weken)

---

Hoeveel uur thuiszorg kreeg u in deze weken gemiddeld?

---

Aantal uur/uren 'huishoudelijke hulp' gemiddeld in de week:

______________________________
(uur/uren)

---

Aantal uur/uren 'verzorging van uzelf' gemiddeld in de week:

______________________________
(uur/uren)

---

Aantal uur/uren 'verpleging' gemiddeld in de week:

______________________________
(uur/uren)

## Medicijnen

Heeft u in de afgelopen 3 maanden medicijnen gebruikt?

◯ Nee
◯ Ja

Welke medicijnen heeft u in de afgelopen 3 maanden gebruikt?
(Met medicijnen bedoelen we alle medicijnen die u hebt gekregen op voorschrift en medicijnen die u hebt gekocht bij de apotheek. U ziet eerst drie voorbeelden.)

Let op: pak de verpakking erbij! Daarop staat hoeveel u per keer moest nemen. En hoe vaak u dat moest doen.

Heeft u meer of minder gebruikt? Vul dan in hoeveel u ook echt gebruikt heeft

| ***Hoe heet het medicijn?*** | ***Hoeveel heeft u per keer genomen?*** *Kijk op de verpakking* | ***Hoe vaak op een dag heeft u dit gedaan?*** *Kijk op de verpakking* | ***Op hoeveel dagen in de afgelopen 3 maanden heeft u het medicijn gebruikt?*** |
|---|---|---|---|
| *voorbeeld 1* Metoprolol (tegen hoge bloeddruk) | Voorbeeld 100mg | Voorbeeld 1 keer | Voorbeeld 90 dagen |
| *voorbeeld 2 Furosemide (plastabletten)* | Voorbeeld 40 mg | Voorbeeld 1 keer | Voorbeeld 26 dagen (2x per week, 13 weken) |
| Voorbeeld 3 Hydrocortison crème | Voorbeeld - | Voorbeeld 1 | Voorbeeld 14 dagen |

Hoe heten de medicijnen? Hoeveel heeft u per keer ingenomen? Hoe vaak op een dag heeft u dit gedaan? Op hoeveel dagen in de afgelopen 3 maanden heeft u dit medicijn gebruikt?
Voorbeeld: Aspirine Voorbeeld 100mg Voorbeeld: 1 keer Voorbeeld: 3 maanden
{imcq_be_q11b2_m1_name} {imcq_be_q11b2_m1_dose}

{imcq_be_q11b2_m1_freq} {imcq_be_q11b2_m1_much}
{imcq_be_q11b2_m2_name} {imcq_be_q11b2_m2_dose} {imcq_be_q11b2_m2_freq} {imcq_be_q11b2_m2_much}
{imcq_be_q11b2_m3_name} {imcq_be_q11b2_m3_dose} {imcq_be_q11b2_m3_freq} {imcq_be_q11b2_m3_much}
{imcq_be_q11b2_m4_name} {imcq_be_q11b2_m4_dose} {imcq_be_q11b2_m4_freq} {imcq_be_q11b2_m4_much}
{imcq_be_q11b2_m5_name} {imcq_be_q11b2_m5_dose} {imcq_be_q11b2_m5_freq} {imcq_be_q11b2_m5_much}
{imcq_be_q11b2_m6_name} {imcq_be_q11b2_m6_dose} {imcq_be_q11b2_m6_freq} {imcq_be_q11b2_m6_much}
{imcq_be_q11b2_m7_name} {imcq_be_q11b2_m7_dose} {imcq_be_q11b2_m7_freq} {imcq_be_q11b2_m7_much}
{imcq_be_q11b2_m8_name} {imcq_be_q11b2_m8_dose} {imcq_be_q11b2_m8_freq} {imcq_be_q11b2_m8_much}
{imcq_be_q11b2_m9_name} {imcq_be_q11b2_m9_dose} {imcq_be_q11b2_m9_freq} {imcq_be_q11b2_m9_much}
{imcq_be_q11b2_m10_name} {imcq_be_q11b2_m10_dose} {imcq_be_q11b2_m10_freq}
{imcq_be_q11b2_m10_much}

## Ziekenhuis of ambulance

Bent u in de afgelopen 3 maanden in het ziekenhuis of op spoed geweest? (Zowel met verblijf in het ziekenhuis als zonder).

◯ Nee
◯ Ja

Hoe vaak bent u in de afgelopen 3 maanden in het ziekenhuis of op spoed geweest?

◯ Geen enkele keer
◯ ... {imcq_be_q12b1} keer

Hoe vaak bent u de afgelopen 3 maanden met een ambulance naar het ziekenhuis gebracht? Een andere naam voor ambulance is ziekenwagen.

◯ Geen enkele keer
◯ ... {imcq_be_q13a} keer

Had u in de afgelopen 3 maanden een afspraak bij de polikliniek van het ziekenhuis? Het gaat om afspraken voor uzelf met een dokter. Bijvoorbeeld met cardioloog, reumatoloog of neuroloog.

◯ Nee
◯ Ja

Bij welke soort dokter bent u in het ziekenhuis geweest? (Bv: cardioloog) Hoe vaak bent u in de afgelopen 3 maanden bij deze dokter geweest? (bv: 2 keer)
{imcq_be_q14b_dr1_name} {imcq_be_q14b_dr1_freq}
{imcq_be_q14b_dr2_name} {imcq_be_q14b_dr2_freq}
{imcq_be_q14b_dr3_name} {imcq_be_q14b_dr3_freq}
{imcq_be_q14b_dr4_name} {imcq_be_q14b_dr4_freq}
{imcq_be_q14b_dr5_name} {imcq_be_q14b_dr5_freq}
{imcq_be_q14b_dr6_name} {imcq_be_q14b_dr6_freq}

Bent u in de afgelopen 3 maanden overdag in het ziekenhuis geweest voor een behandeling? U bleef dus niet slapen. U kwam bijvoorbeeld voor bloedtransfusie, nierdialyse of chemokuur.

◯ Nee
◯ Ja

Voor welke soort behandeling(en) was dit? Was dit voor meer dan 1 soort behandeling, vul dan alle soorten behandelingen in.

Hoeveel keer moest u in de afgelopen 3 maanden voor (elk van) deze behandelingen naar het ziekenhuis?

## Dagbehandeling

Bent u in de afgelopen 3 maanden ergens geweest voor een behandeling overdag? U bleef dus niet slapen. U ging bijvoorbeeld naar een

- Woon-/zorgcentrum
- Revalidatiecentrum
- Psychiatrische instelling
- Andere instelling

◯ Nee
◯ Ja

Wat voor instelling was dit? Kruis het goede antwoord aan.

U kunt meer dan 1 hokje aanduiden.

☐ Woon-/zorgcentrum
☐ Revalidatiecentrum
☐ Psychiatrische instelling
☐ Een andere instelling, namelijk: {imcq_be_q16b2}

Hoe vaak moest u hier in de afgelopen 3 maanden naartoe?

Vul hieronder voor iedere instelling in hoe vaak u er bent geweest.

Aantal keer dat u in de afgelopen 3 maanden naar het woon-/zorgcentrum moest:

______________________________
(keer)

Aantal keer dat u in de afgelopen 3 maanden naar het revalidatiecentrum moest:

______________________________
(keer)

Aantal keer dat u in de afgelopen 3 maanden naar de psychiatrische instelling moest:

______________________________

Aantal keer dat u in de afgelopen 3 maanden naar 'een andere' instelling moest:

______________________________
(keer)

Heeft u in de afgelopen 3 maanden weleens in het ziekenhuis gelegen?

U moest dus blijven slapen. Bijvoorbeeld omdat u geopereerd was en niet meteen naar huis kon.

◯ Nee
◯ Ja

Hoe vaak heeft u in de afgelopen 3 maanden in het ziekenhuis gelegen?

◯ Geen enkele keer
◯ ... {imcq_be_q17b2} keer

Hoe lang heeft u in het ziekenhuis gelegen?

Heeft u meer dan 1 keer in het ziekenhuis gelegen in de afgelopen 3 maanden? Tel dan alle dagen bij elkaar op.

◯ Geen enkele keer
◯ ... {imcq_be_q17c2} keer

Moest u in de afgelopen 3 maanden ergens anders blijven slapen voor uw gezondheid?

Bijvoorbeeld in een

- Woon-/zorgcentrum
- Revalidatiecentrum
- Psychiatrische instelling
- Andere instelling

◯ Nee
◯ Ja

Wat voor instelling was dit? U kunt meer dan 1 hokje aanduiden.

☐ woon-/zorgcentrum
☐ revalidatiecentrum
☐ psychiatrische instelling
☐ een andere instelling, namelijk {imcq_be_q18b2}

Hoe lang bent u in deze instelling geweest?

Vul hieronder voor iedere instelling in hoe lang u er bent geweest.

Aantal dagen dat u in de afgelopen 3 maanden in het woon-/zorgcentrum bent geweest:

______________________________
(dag/dagen)

Aantal dagen dat u in de afgelopen 3 maanden in het revalidatiecentrum bent geweest:

______________________________
(dag/dagen)

Aantal dagen dat u in de afgelopen 3 maanden in de psychiatrische instelling bent geweest:

______________________________
(dag/dagen)

Aantal dagen dat u in de afgelopen 3 maanden in 'de andere' instelling bent geweest:

______________________________
(dag/dagen)

## Ondersteuning in het dagelijks leven

Heeft u in de afgelopen 3 maanden hulp gekregen van een familielid of een bekende vanwege uw lichamelijke of psychische problemen? Bijvoorbeeld:

- Huishoudelijke hulp (bijvoorbeeld: stofzuigen, bed opmaken, boodschappen doen, klaarmaken van eten en drinken, verzorgen van kinderen)
- Verzorging van uzelf (bijvoorbeeld: hulp bij douchen of aankleden, hulp bij het eten en drinken of het geven van medicijnen)
- Praktische hulp (bijvoorbeeld: ondersteuning bij wandelen, het maken van uitstapjes of bezoekjes aan bekenden, bezoeken aan de huisarts of het ziekenhuis, het regelen van hulp of het regelen van financiële zaken)

◯ Nee
◯ Ja

---

Wat voor hulp van familieleden of bekenden heeft u gehad in de afgelopen 3 maanden?

U kunt meer dan 1 hokje aankruisen.

☐ Huishoudelijke hulp (bijvoorbeeld: stofzuigen, bed opmaken, boodschappen doen, klaarmaken van eten en drinken, verzorgen van kinderen)
☐ Verzorging van uzelf (bijvoorbeeld: hulp bij douchen of aankleden, hulp bij het eten en drinken of het geven van medicijnen)
☐ Praktische hulp (bijvoorbeeld: ondersteuning bij wandelen, het maken van uitstapjes of bezoekjes aan bekenden, bezoeken aan de huisarts of het ziekenhuis, het regelen van hulp of het regelen van financiële zaken)

---

Hoeveel weken heeft u deze hulp gehad? Tel alle weken in de afgelopen 3 maanden bij elkaar op.

---

Aantal weken dat u afgelopen 3 maanden huishoudelijke hulp kreeg.

______________________________
(In weken)

---

Aantal weken dat u in de afgelopen 3 maanden hulp kreeg voor de verzorging van uzelf.

______________________________
(In weken)

---

Aantal weken dat u in de afgelopen 3 maanden hulp kreeg voor praktische hulp.

______________________________
(In weken)

---

Hoeveel uur hulp kreeg u in deze weken gemiddeld?

---

Gemiddeld aantal uren in de week voor huishoudelijk hulp.

______________________________
(Uur/uren)

---

Gemiddeld aantal uren in de week voor verzorging van uzelf.

______________________________
(Uur/in uren)

Gemiddeld aantal uren in de week voor praktische hulp.

__________________________________
(uur/uren)

Welke wijze van vervoer heeft u gebruikt om van huis naar het ziekenhuis te gaan?

◯ Niet van toepassing
◯ Te voet
◯ Fiets
◯ Auto
◯ Openbaar vervoer
◯ Taxi
◯ anders, namelijk ... {imcq_be_q20a2}

Wat was de enkele reisafstand in kilometer tussen uw huis en het ziekenhuis?

__________________________________

Dit was de laatste vraag van deze vragenlijst.

Heeft u vragen of opmerkingen?
Misschien heeft u nog vragen of opmerkingen? Schrijft u deze dan hieronder op.

Hartelijk dank!

__________________________________

# Vragenlijst gezondheid en gezondheidsgerelateerde levenskwaliteit

**Instructies:**

**De onderstaande vragen hebben betrekking op levenskwaliteit.**

**Vink onder elke titel het ENE vakje aan dat het best uw gezondheid VANDAAG beschrijft.**

**Gelieve alle vragen te beantwoorden.**

1) Mobiliteit

◯ Ik heb geen problemen met rondwandelen
◯ Ik heb een beetje problemen met rondwandelen
◯ Ik heb matige problemen met rondwandelen
◯ Ik heb ernstige problemen met rondwandelen
◯ Ik ben niet in staat om rond te wandelen

2) Zelfzorg

◯ Ik heb geen problemen met mijzelf te wassen of aan te kleden.
◯ Ik heb een beetje problemen met mijzelf te wassen of aan te kleden.
◯ Ik heb matige problemen met mijzelf te wassen of aan te kleden.
◯ Ik heb ernstige problemen met mijzelf te wassen of aan te kleden.
◯ Ik ben niet in staat mijzelf te wassen of aan te kleden.

3) Dagelijkse activiteiten (Bijv. werk, studie, huishouden, gezins- en vrijetijdsactiviteiten)

◯ Ik heb geen problemen met het uitvoeren van mijn dagelijkse activiteiten.
◯ Ik heb een beetje problemen met het uitvoeren van mijn dagelijkse activiteiten.
◯ Ik heb matige problemen met het uitvoeren van mijn dagelijkse activiteiten.
◯ Ik heb ernstige problemen met het uitvoeren van mijn dagelijkse activiteiten.
◯ Ik ben niet in staat mijn dagelijkse activiteiten uit te voeren.

4) Pijn / Ongemak

◯ Ik heb geen pijn of ongemak.
◯ Ik heb een beetje pijn of ongemak.
◯ Ik heb matige pijn of ongemak.
◯ Ik heb ernstige pijn of ongemak.
◯ Ik heb extreme pijn of ongemak.

5) Angst / Depressie

◯ Ik ben niet angstig of depressief.
◯ Ik ben een beetje angstig of depressief.
◯ Ik ben matig angstig of depressief.
◯ Ik ben erg angstig of depressief.
◯ Ik ben extreem angstig of depressief.

6) We willen weten hoe goed of slecht uw gezondheid VANDAAG is.

De meetschaal (te vergelijken met een thermometer) is genummerd van 0 tot 100.
- 100 staat voor de beste gezondheid die u zich kunt voorstellen.
- 0 staat voor de slechtste gezondheid die u zich kunt voorstellen.

Verplaats de slider op de meetschaal om aan te geven hoe uw gezondheid VANDAAG is.

| 0: slechtste gezondheid die u zich kan voorstellen | 50 | 100: beste gezondheid die u zich kan voorstellen |
|---|---|---|

*(Place a mark on the scale above)*
