# Supplementary material for: Economic evaluation of a lifestyle intervention for individuals with overweight or obesity suffering from chronic low back pain (the BO2WL trial): a protocol for a health economic analysis
Source: BMJ Open. 2025 Jun 20;15(6):e098272. doi: 10.1136/bmjopen-2024-098272 (PMC12184353; doi:10.1136/bmjopen-2024-098272)
Supplement: online supplemental file 2 [file bmjopen-15-6-s002.pdf]

institute for
Medical Technology Assessment

Medical Consumption Questionnaire

Productivity & Health Research Group

SAMPLE
NOT FOR DATA COLLECTION

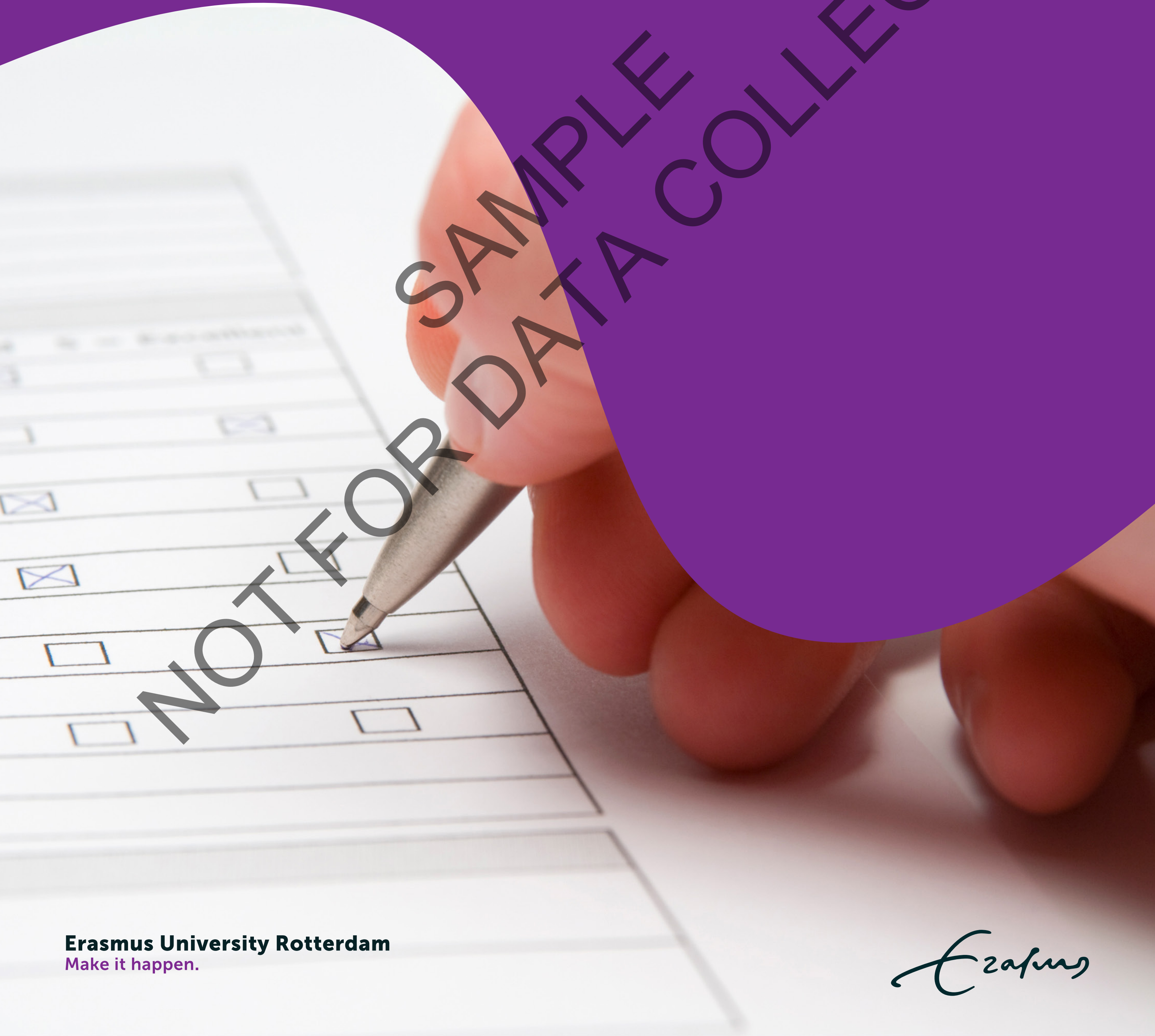

Erasmus University Rotterdam
Make it happen.

Questionnaire about your use of care

SAMPLE
NOT FOR DATA COLLECTION

Erasmus Universiteit Rotterdam
iMTA

Researchers call this questionnaire the iMTA MCQ (iMCQ).

July 2018 version

Comment

Please read this first!

Who is this questionnaire for?
This questionnaire is for you. There are various possibilities:

- You have received the list from your general practitioner or in the hospital.
- You have received the list by mail and your name is on the envelope.

What is the questionnaire about?
The questionnaire is about your use of care in the past 3 months. We start with general questions. For example about your gender and date of birth. Then we ask questions about your use of care.

How long does it take to fill in the list?
It takes about 20 minutes to fill in the list.

How do you have to fill in the list?

- Start with the first question and follow the numbering.
- For each question, tick 1 box, except if the question states that you can tick more than 1 box.
- For some questions you can enter a number or something else on the dotted line.
- You can not give wrong answers.

Do you want to change an answer?

- Strike through the old answer.
- Tick a new answer.
- Put an arrow for the new answer.

☒ ~~old answer~~
→ ☒ new answer

SAMPLE
NOT FOR DATA COLLECTION

What happens to your answers?
Your answers are used for research. Only the researchers will see your answers. So no one else.

Your data will be anonymised. This means that they are not traceable to you. The researchers do not tell anyone that you participated in the study.

Can not you fill in the list yourself?
If you cannot fill in the list yourself, someone might be able to help you. For example a family member.

Thank you for filling in the list for us!

SAMPLE
NOT FOR DATA COLLECTION

General questions

Question A1. On what date did you fill in this questionnaire?

Day		Month		Year			

Question A2. What is your date of birth?

Day		Month		Year			

Question A3. What is your gender?

☐ Male
☐ Female

Question A4. What is the highest level of education you have completed? Find your highest level of education and tick the box for it.

☐ I have not finished school or training
☐ Primary school or elementary school
☐ Junior vocational education

☐ Lower general secondary school
☐ Intermediate vocational eduction
☐ Higher general secondary education

☐ School for higher vocational education
☐ University
☐ I have completed another training, namely ....

……………………………………………………………………….

SAMPLE
NOT FOR DATA COLLECTION

Question A5. What do you do in daily life? Tick what you do most of the time.

☐ I am in school, I am studying
☐ I work in paid employment
☐ I am an independent entrepreneur
☐ I am a housewife, a houseman

☐ I am unemployed
☐ I am disabled, for... %
☐ I am retired or pre-retired
☐ I'm doing something else, namely ....

…………………………………………………………………………

SAMPLE
NOT FOR DATA COLLECTION

Questions about healthcare use

Comment
We would like to know which doctors you have consulted in the past 3 months. It is about consultations for yourself. Other healthcare providers also count. For example, the physiotherapist.

Which consultations count?

- Control visits
- Appointments because you had a physical or psychological complaint
- Appointments where the doctor came to your home
- Telephone appointments
- Phone calls with the recipe line

Which consultations do not count?

- Appointments for another person, for example for your child
- Telephone calls to make an appointment

Are you unsure about the exact number of consultations? Please fill in how many consultations you have had approximately.

Question 1a. Have you consulted a general practitioner or nurse practitioner in the past 3 months?

☐ No
☐ Yes

Have you ticked "Yes"? Then answer question 1b.
Otherwise, continue with question 2.

Question 1b. How many appointments did you have with your GP and/or nurse practitioner in the past 3 months?

☐ ...... appointments with a doctor
☐ ...... appointments with a nurse practitioner

SAMPLE
NOT FOR DATA COLLECTION

Question 2. How many appointments did you have with a social worker in the past 3 months?

☐ No appointment
☐ …… appointments

Question 3. How many appointments did you have with a physiotherapist in the past 3 months? Or with a Caesar therapist, therapist Mensendieck or a manual therapist? Add up all appointments with these therapists.

☐ No appointment
☐ …… appointments

Question 4. How many appointments did you have with an occupational therapist in the past 3 months?

☐ No appointment
☐ …… appointments

Question 5. How many appointments did you have with a speech therapist in the past 3 months?

☐ No appointment
☐ …… appointments

Question 6. How many appointments did you have with a dietitian in the past 3 months?

☐ No appointment
☐ …… appointments

SAMPLE NOT FOR DATA COLLECTION

Question 7. How many appointments did you have with a homeopath in the past 3 months? Or with an acupuncturist? Add up all appointments with these healthcare providers.

- ☐ No appointment
- ☐ …… appointments

Question 8. How many appointments did you have with a psychologist in the past 3 months? Or with a psychotherapist or psychiatrist? Add up all appointments with these healthcare providers.

- ☐ No appointment
- ☐ …… appointments

Question 9 How many appointments did you have with the company doctor in the past 3 months?

- ☐ No appointment
- ☐ …… appointments

Question 10a. Have you received home care in the past 3 months?

- ☐ No
- ☐ Yes

Have you ticked "Yes"? Then answer questions 10b through 10d.
Otherwise, continue with question 11.

SAMPLE
NOT FOR DATA COLLECTION

Question 10b. What kind home care have you had in the past 3 months?
You can tick more than 1 box.

- ☐ Housekeeping and domestic help
  example: vacuuming, making bed, going for daily groceries

- ☐ Personal care
  example: help with bathing or dressing

- ☐ Nursing
  example: putting on a bandage, administering medication, measuring blood pressure

Question 10c. How many weeks did you have this home care? Count up all weeks in the past 3 months. *Note: a period of 3 months counts* *13 weeks*.

Domestic help: ...... weeks in the past 3 months

Personal care: ...... weeks in the past 3 months

Nursing: ...... weeks in the past 3 months

Question 10d. How many hours of home care did you receive on average in these weeks?

Domestic help: on average ...... hours a week

Personal care: on average ...... hours a week

Nursing: on average ...... hours a week

SAMPLE
NOT FOR DATA COLLECTION

Question 11a. Did you take any medication in the past 3 months?

☐ No
☐ Yes

Have you ticked "Yes"? Then fill in question 11b listing which medications you used and how much.
Otherwise, continue with question 12.

Question 11b. What medication did you take in the past 3 months? By medication we mean all drugs that you have received on prescription and medication that you have bought at the pharmacy or drugstore. There are three examples below.

Pay attention: look at the package! It shows how much you had to take at each time. And how often you had to do so per day. ***Have you used more or less? Then enter how much you have actually used.***

What is the name of the medicine?	***How much did you take at each time?*** *Look at the packaging*	***How many times did you take this per day?*** *Look at the packaging*	***On how many days in the past 3 months have you used the medication?***
example 1<br><br>Metoprolol (against high blood pressure)	*example*<br><br>100 mg	*example*<br><br>1 time	*example*<br><br>90 days
example 2<br><br>Furosemide (diuretic)	*example*<br><br>40 mg	*example*<br><br>1 time	*example*<br><br>26 days (2 x per week, 13 weeks)
example 3<br><br>Hydrocortisone cream	*example*<br><br>-	*example*<br><br>1	*example*<br><br>14 days
……………………….	…………………	………………….	…………………..
……………………….	…………………	………………….	…………………..
……………………….	…………………	………………….	…………………..
……………………….	…………………	………………….	…………………..
……………………….	…………………	………………….	…………………..
……………………….	…………………	………………….	…………………..
……………………….	…………………	………………….	…………………..
……………………….	…………………	………………….	…………………..

NOT FOR DATA COLLECTION
SAMPLE

Question 12. How many times did you visit the emergency room of a hospital for the past 3 months?

☐ Not once
☐ …… times

Question 13. How many times have you been taken to the hospital with an ambulance in the past 3 months?

☐ Not once
☐ …… times

Question 14a. Did you have an appointment at the outpatient clinic of the hospital in the past 3 months? It is about appointments for yourself with a doctor. For example with the cardiologist, rheumatologist or neurologist.

☐ No
☐ Yes

Have you ticked "Yes"? Then fill in question 14b the types of doctors you have visited. And how often. There is an example in the first row.
Otherwise, continue with question 15.

SAMPLE
NOT FOR DATA COLLECTION

Question 14b. Which types of doctors have you been to in hospital for the past 3 months? And how often?

Which type of doctor did you visit in the hospital?	***How often have you been with this doctor in the past 3 months?***
example<br>cardiologist	*example*<br>2 times
………………………………………………	……times
………………………………………………	……times
………………………………………………	……times
………………………………………………	……times
………………………………………………	……times
………………………………………………	……times
………………………………………………	……times

Question 15a. Did you visit the hospital for day care treatment during the past 3 months? So you did not stay overnight. Examples of day care treatments are blood transfusions, renal dialysis or a chemo course.

☐ No
☐ Yes

Have you ticked "Yes"? Then answer questions 15b and 15c.
Otherwise, continue with question 16.

Question 15b. For what kind of treatment was this? Was this for more than one type of treatment? Then enter all types of treatments.

Treatment 1:. ....

Treatment 2:. ....

Treatment 3:. ....

SAMPLE
NOT FOR DATA COLLECTION

Question 15c. How many times did you have to go to the hospital for these treatments in the past 3 months?

...... times for treatment 1

...... times for treatment 2

...... times for treatment 3

Question 16a. Did you go elsewhere for a day care treatment in the past 3 months? So you did not stay overnight. For example, you went to the day care treatment centre of a residential/care centre or a psychiatric institution. Or to the day care treatment centre of a rehabilitation centre.

☐ No
☐ Yes

Have you ticked "Yes"? Then answer questions 16b and 16c.
Otherwise, continue with question 17.

Question 16b. What kind of institution was this? Tick the correct answer. You can tick more than 1 box.

☐ Residential care centre or nursing home
☐ Rehabilitation centre
☐ Mental health institution
☐ Another institution, namely ....

…………………………………………………………………………

SAMPLE
NOT FOR DATA COLLECTION

Question 16c. How many times did you have to go here in the past 3 months? Have you ticked more than one box in question 16b? Then enter the number of times you have been there for each institution below.

To a residential care or nursing home: ...... times in the past 3 months

To the rehabilitation centre: ...... times in the past 3 months

To the mental health institution: ...... times in the past 3 months

To the other institution: ...... times in the past 3 months

Question 17a. Have you been admitted to a hospital for inpatient care in the past 3 months? So you had to stay overnight. For example, because you had surgery and could not go home immediately.

☐ No
☐ Yes

Have you ticked "Yes"? Then answer questions 17b and 17c.
Otherwise, continue with question 18.

Question 17b. How often have you been admitted to the hospital for inpatient care in the past 3 months?

...... times in the past 3 months

Question 17c. How long have you stayed in the hospital? Have you been in the hospital more than once in the past 3 months? Then add all the days together.

...... days in total in the past 3 months

SAMPLE NOT FOR DATA COLLECTION

Question 18a. Have you been admitted elsewhere for your health in the past 3 months? For example in a residential/care centre, psychiatric institution or rehabilitation centre.

☐ No
☐ Yes

Have you ticked "Yes"? Then answer questions 18b and 18c.
Otherwise, go to the end of the questionnaire.

Question 18b. What kind of institution was this? You can tick more than 1 box.

☐ Residential care centre or nursing home
☐ Rehabilitation centre
☐ Mental health institution
☐ Another institution, namely ....

…………………………………………………………………………

Question 18c. How long have you been in this institution? Have you ticked more than one box in question 18b? Then fill in for each setting below how long you have been there.

Have you been anywhere more than 1 time in the past 3 months? Then add all the days together.

In the residential care centre or nursing home: ...... days in the past 3 months

In the rehabilitation centre: ...... days in the past 3 months

In the mental health institution: ...... days in the past 3 months

In the other institution: ...... days in the past 3 months

That was the last question.

NOT FOR DATA COLLECTION SAMPLE

Do you have questions or comments?
If you have any questions or comments, list them here.

……………………………………………………………………………………………
……………………………………………………………………………………………
……………………………………………………………………………………………
……………………………………………………………………………………………
……………………………………………………………………………………………
……………………………………………………………………………………………
……………………………………………………………………………………………
……………………………………………………………………………………………
……………………………………………………………………………………………
……………………………………………………………………………………………
……………………………………………………………………………………………
……………………………………………………………………………………………
……………………………………………………………………………………………
……………………………………………………………………………………………
……………………………………………………………………………………………
……………………………………………………………………………………………
……………………………………………………………………………………………
……………………………………………………………………………………………

NOT FOR DATA COLLECTION
SAMPLE

What should you do with the completed questionnaire?
If everything is filled in correctly, place the questionnaire in the envelope. There is no need to use a stamp. Please send it back to us quickly, before xxx.

If you have lost the envelope, you can send the questionnaire in another envelope without a stamp to:

x x x
x x x
x x x

Thank you!

Optional questions

> **<u>For the researcher</u>:** questions 19 and 20 can be added to the questionnaire if you want to measure the costs of patients and family in a social perspective. Only do this if these questions are relevant!

Question 19a. Have you received help from a family member or acquaintance due to your physical or psychological problems in the past 3 months?

- ☐ No
- ☐ Yes

Have you ticked "Yes"? Then answer questions 19b through 19d.
Otherwise, continue with question 20.

Question 19b. What kind of help from family members or acquaintances have you had in the past 3 months? You can tick more than 1 box.

- ☐ Domestic help
  example: vacuuming, making bed, shopping, preparing food and drinks, taking care of children

- ☐ Personal care
  example: help with showering or dressing, help with eating and drinking or giving medicines

- ☐ Practical help
  example: support with walking, making trips or visits to acquaintances, visits to the general practitioner or the hospital, arranging help or arranging financial affairs

NOT FOR DATA COLLECTION
SAMPLE

Question 19c. How many weeks did you have this home care? Count up all the weeks in the past 3 months. *Note: a period of 3 months counts as 13 weeks*.

Domestic help: ... ... weeks in the past 3 months

Personal care: ... ... weeks in the past 3 months

Practical help: ... ... weeks in the past 3 months

Question 19d. How many hours of home care did you receive on average in these weeks?

Domestic help: on average ...... hours a week

Personal care: on average ...... hours a week

Practical help: on average ...... hours a week

Question 20a. What type of transport have you used to go from home to the hospital?

- ☐ Not applicable
- ☐ Pedestrian
- ☐ Bicycle
- ☐ Car

- ☐ Public transport
- ☐ Cab
- ☐ Otherwise, namely

……………………………………………………………………………

Question 20b. What was the one-way distance between your home and the hospital?

This distance was: ...... kilometres

SAMPLE
NOT FOR DATA COLLECTION

Institute for
Medical
Technology
Assessment

Productivity Cost Questionnaire

Productivity & Health Research Group

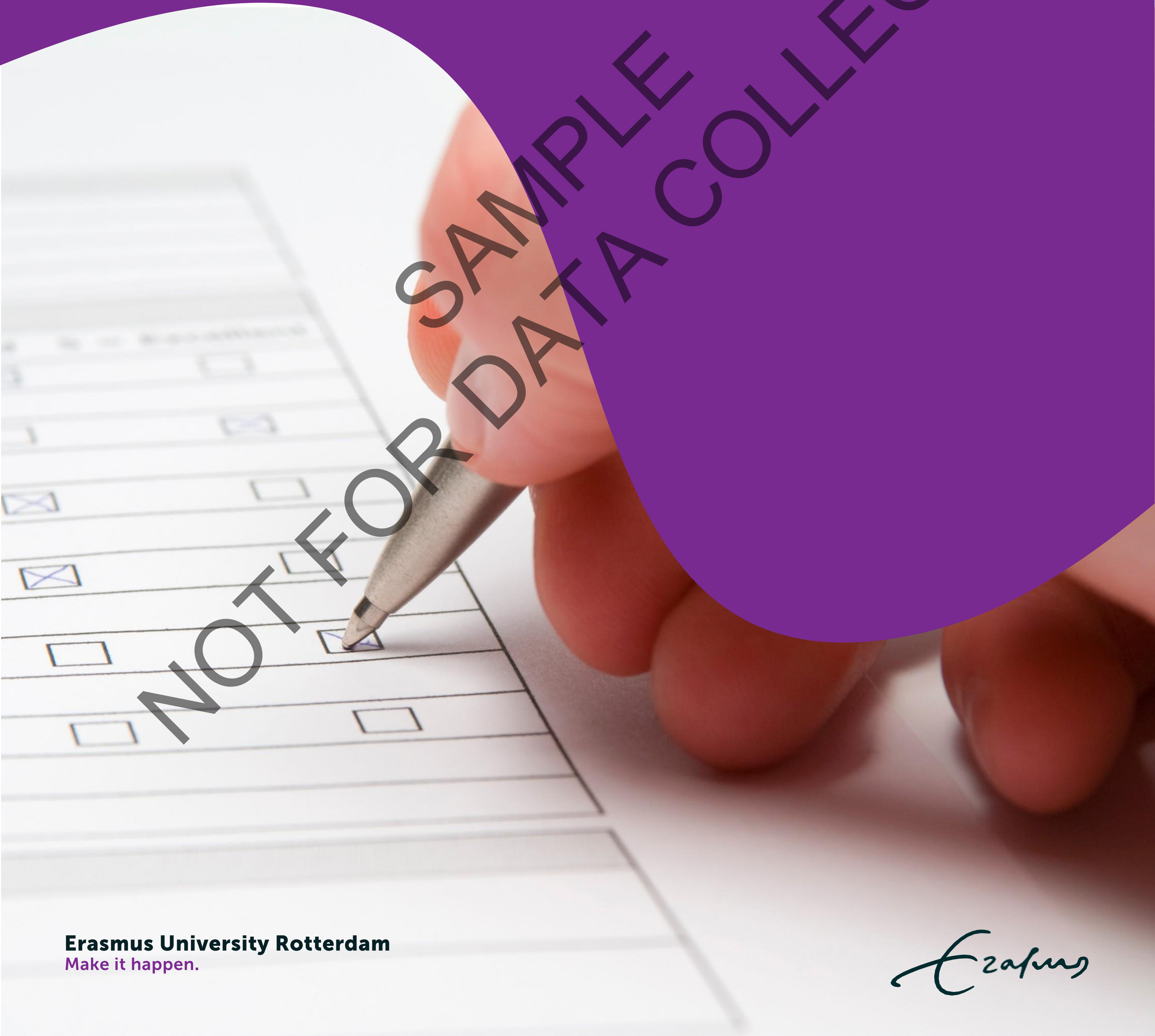

SAMPLE
NOT FOR DATA COLLECTION

Erasmus University Rotterdam
Make it happen.

Questionnaire about your health and work

SAMPLE
NOT FOR DATA COLLECTION

Researchers call this questionnaire the iMTA PCQ.

US version

Explanatory notes

Please read this first!

Who is this questionnaire for?
This questionnaire is for you. There are various possibilities:

- You received the questionnaire from your primary care physician or in the hospital.
- You received the questionnaire by mail and your name is on the envelope.

Are you unable to complete the questionnaire yourself?
If you are unable to complete the questionnaire yourself, perhaps someone can help you. A member of your family, for example.

What is the questionnaire about?
The questionnaire is about your health and work in the last 4 weeks. We will start with general questions. For example, about your gender and date of birth.

How long does it take to complete the questionnaire?
It takes roughly 10 minutes to complete the questionnaire.

How should you complete the questionnaire?

- Start with the first question and follow the numbering.
- Check 1 box for each question, unless the question says that you can check more than 1 box.
- For some questions, you can enter a number or something else on the dotted line.
- There are no wrong answers.

SAMPLE
NOT FOR DATA COLLECTION

Do you want to change an answer?

- Strike through the old answer.
- Enter a check next to a new answer.
- Put an arrow in front of the new answer.

~~☒ old answer~~
→ ☒ new answer

What will happen with your answers?
Your answers will be used for research. Only the researchers will see your answers. So, nobody else will.

The researchers will not write down your name anywhere. And they will not tell anyone that you have taken part in the trial.

It is great that you want to complete the questionnaire for us!

SAMPLE
NOT FOR DATA COLLECTION

General questions

Question A1. On what date are you completing this questionnaire?

month day year

☐☐ ☐☐ ☐☐☐☐

Question A2. What is your date of birth?

month day year

☐☐ ☐☐ ☐☐☐☐

Question A3. What is your gender?

☐ Male
☐ Female

Question A4. What is the highest level of education you have completed? Look for your highest level of education and check the relevant box.

☐ I have not completed school or any education
☐ Elementary School
☐ High School Diploma
☐ Sub-bachelor or Vocational Diploma or Certificate
☐ Associate Degree
☐ Bachelor's Degree
☐ First Professional Degree
☐ Post-bachelor's Diploma/Certificate
☐ Master's Degree
☐ Doctorate or Advanced Professional Degree
☐ I completed a different course, namely …………

………………………………………………………………………

SAMPLE
NOT FOR DATA COLLECTION

Question A5. What is your occupation? Check the box for what best describes your primary occupation.

- ☐ I am at school, I study
- ☐ I am in paid employment
- ☐ I am self-employed
- ☐ I am a housewife/househusband

- ☐ I am unemployed
- ☐ I am disabled for working, for, … % (bodily percentage)
- ☐ I am retired or have taken early retirement
- ☐ I do something else, namely …………………………………..

…………………………………………………………………………

Question A6. Do you have paid work?

- ☐ No
- ☐ Yes

SAMPLE
NOT FOR DATA COLLECTION

You will first have questions about your job. So, about work for which you are paid. *Do you not have a paid job?* Then continue with question 10. First, read the explanatory notes above question 10.

Question 1. What is your occupation?

....

Question 2. How many hours a week do you work? Add together all the hours for which you are paid.

…… hours

Question 3. How many days a week do you work?

…… days

Question 4. Have you been absent from your work in the last four weeks because you were sick?

☐ No
☐ Yes, I was absent for….. days
(*Only count the working days in the last 4 weeks)*

Have you checked the "Yes" box? Then answer question 5.
Otherwise continue with question 7.

SAMPLE
NOT FOR DATA COLLECTION

Question 5. Have you been absent from your work because of being sick for longer than the entire period of 4 weeks? This refers to an uninterrupted period of absence from work.

☐ No
☐ Yes

Have you checked the “Yes” box? Then answer question 6.
Otherwise continue from question 7.

Question 6. When did you call in sick?

month		day		year			

Continue with question 10. *First, read the explanatory notes above question 10*.

Question 7. Have there been days in the last 4 weeks when you worked but suffered from physical or psychological problems during your work?

☐ No
☐ Yes

Have you checked the “Yes” box? Then answer questions 8 and 9.
Otherwise continue with question 10. *First, read the explanatory notes above question 10.*

Question 8. On how many working days have you suffered from physical or psychological problems during your work? Just count the working days in the last 4 weeks.

…… working days

SAMPLE
NOT FOR DATA COLLECTION

Question 9. On the days when you were suffering from problems, perhaps you were not able to do as much work as normal. On those days, how much work could you do on average? Look at the numbers below. A 10 means that you were able to do just as much as normal on those days. A 0 means that you were not able to do anything on those days. Circle the right number.

I was not able to do anything on those days					I could do around half					I was able to do just as much as normal
0	1	2	3	4	5	6	7	8	9	10

Explanatory notes

You may also suffer from your physical or psychological problems during unpaid work. Sometimes you can do less as a result. For example, you cannot care properly for the children or do volunteer work. Or you may not be able shop or do gardening. That is what the next questions are about.

Question 10. Have there been any days on which you were able to do less unpaid work because of your physical or psychological problems? This relates to days in the last 4 weeks.

☐ No
☐ Yes

Have you checked the "Yes" box? Then answer questions 11 and 12.
Otherwise go to the end of the questionnaire.

Question 11. On how many days was this the case? Only count the days in the last four weeks.

…. days

SAMPLE NOT FOR DATA COLLECTION

Question 12. Suppose that someone, for example your partner, a family member or an acquaintance, helped you on these days. And did all that unpaid work for you, that you could not do. How many hours, on average, was that person busy with it on these days?

On average …… hours on these days

That was the last question.

Do you have questions or comments?

Perhaps you have some more questions or comments? If so, please enter them below.

……………………………………………………………………………………………
……………………………………………………………………………………………
……………………………………………………………………………………………
……………………………………………………………………………………………
……………………………………………………………………………………………
……………………………………………………………………………………………
……………………………………………………………………………………………
……………………………………………………………………………………………
……………………………………………………………………………………………
……………………………………………………………………………………………
……………………………………………………………………………………………
……………………………………………………………………………………………

What should you do with the completed questionnaire?

Have you filled in everything? Then put the questionnaire in the envelope. You do not need to put a stamp on it. Please mail the envelope soon. In any event, before xxx.

Have you lost the envelope? In that case, you may send the questionnaire in a different envelope; no stamp required, to:

xxx
xxx
xxx

Thank you very much!

SAMPLE
NOT FOR DATA COLLECTION

Health Questionnaire

English version for the UK

Sample

UK (English) © 2009 EuroQol Group EQ-5D™ is a trade mark of the EuroQol Group

Under each heading, please tick the ONE box that best describes your health TODAY.

MOBILITY

I have no problems in walking about ❑

I have slight problems in walking about ❑

I have moderate problems in walking about ❑

I have severe problems in walking about ❑

I am unable to walk about ❑

SELF-CARE

I have no problems washing or dressing myself ❑

I have slight problems washing or dressing myself ❑

I have moderate problems washing or dressing myself ❑

I have severe problems washing or dressing myself ❑

I am unable to wash or dress myself ❑

USUAL ACTIVITIES *(e.g. work, study, housework, family or leisure activities)*

I have no problems doing my usual activities ❑

I have slight problems doing my usual activities ❑

I have moderate problems doing my usual activities ❑

I have severe problems doing my usual activities ❑

I am unable to do my usual activities ❑

PAIN / DISCOMFORT

I have no pain or discomfort ❑

I have slight pain or discomfort ❑

I have moderate pain or discomfort ❑

I have severe pain or discomfort ❑

I have extreme pain or discomfort ❑

ANXIETY / DEPRESSION

I am not anxious or depressed ❑

I am slightly anxious or depressed ❑

I am moderately anxious or depressed ❑

I am severely anxious or depressed ❑

I am extremely anxious or depressed ❑

Sample

UK (English) © 2009 EuroQol Group EQ-5D™ is a trade mark of the EuroQol Group

- We would like to know how good or bad your health is TODAY.
- This scale is numbered from 0 to 100.
- 100 means the best health you can imagine.
  0 means the worst health you can imagine.
- Mark an X on the scale to indicate how your health is TODAY.
- Now, please write the number you marked on the scale in the box below.

YOUR HEALTH TODAY = ☐

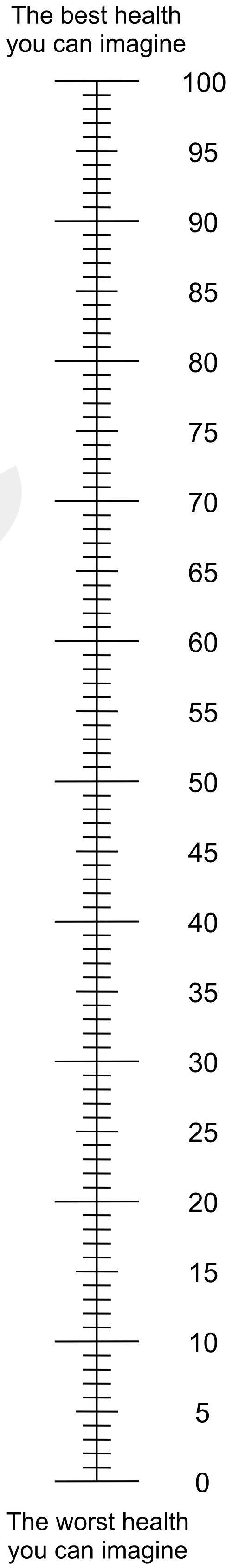

Sample

UK (English) © 2009 EuroQol Group EQ-5D™ is a trade mark of the EuroQol Group
