# Supplementary material for: Economic evaluation of a lifestyle intervention for individuals with overweight or obesity suffering from chronic low back pain (the BO2WL trial): a protocol for a health economic analysis
Source: BMJ Open. 2025 Jun 20;15(6):e098272. doi: 10.1136/bmjopen-2024-098272 (PMC12184353; doi:10.1136/bmjopen-2024-098272)
Supplement: online supplemental file 3 [file bmjopen-15-6-s003.pdf]

Medical Consumption Questionnaire

Bitte füllen Sie die folgende Umfrage aus.Vielen Dank!

Medical Consumption Questionnaire

Bitte lesen Sie dies zuerst!

Worum geht es in dem Fragebogen?
In dem Fragebogen geht es um Ihre Inanspruchnahme von Pflegeleistungen in den letzten 3 Monaten.

Wie lange dauert es, die Liste auszufüllen?
Das Ausfüllen der Liste dauert etwa 20 Minuten.

Wie müssen Sie die Liste ausfüllen?
Beginnen Sie mit der ersten Frage und folgen Sie der Nummerierung.
Kreuzen Sie bei jeder Frage 1 Kästchen an, es sei denn, in der Frage steht, dass Sie mehr als 1 Kästchen ankreuzen können.
Sie können keine falschen Antworten geben.

Was geschieht mit Ihren Antworten?
Ihre Antworten werden für die Forschung verwendet. Nur die Forscher werden Ihre Antworten sehen. Also niemand sonst.

Ihre Daten werden anonymisiert. Das bedeutet, dass sie nicht zu Ihnen zurückverfolgt werden können. Die Forscher sagen niemandem, dass Sie an der Studie teilgenommen haben.

Können Sie die Liste nicht selbst ausfüllen?
Wenn Sie die Liste nicht selbst ausfüllen können, kann Ihnen vielleicht jemand helfen. Zum Beispiel ein Familienmitglied.

Danke, dass Sie die Liste für uns ausfüllen!

Fragen zur Nutzung des Gesundheitswesens
Wir möchten gerne wissen, welche Ärzte Sie in den letzten 3 Monaten konsultiert haben. Es geht um Konsultationen für Sie selbst. Andere Gesundheitsdienstleister zählen auch. Zum Beispiel den Physiotherapeuten.

Welche Konsultationen zählen?
- Kontrollbesuche
- Termine wegen körperlicher oder psychischer Beschwerden
- Termine, bei denen der Arzt zu Ihnen nach Hause kommt
- Telefonische Termine
- Telefongespräche mit der Rezeptleitung

Welche Konsultationen werden nicht gezählt?
- Termine für eine andere Person, zum Beispiel für Ihr Kind
- Telefonanrufe, um einen Termin zu vereinbaren

Sind Sie sich über die genaue Anzahl der Konsultationen unsicher? Bitte geben Sie an, wie viele Konsultationen Sie ungefähr hatten.

Frage 1. Haben Sie in den letzten 3 Monaten eine medizinische Fachperson konsultiert?
Darunter fallen:
- ArztIn für Allgemeinmedizin/HausarztIn
- Medizinische Fachkraft
- SozialarbeiterIn
- PhysiotherapeutIn
- ErgotherapeutIn
- LogopädIn
- ErnährungsberaterIn
- Homöopathen/Akupunkteur
- PsychologIn, PsychotherapeutIn, PsychaterIn
- Betriebsarzt

◯ Nein
◯ Ja

Frage 1a. Haben Sie in den letzten 3 Monaten einen Arzt für Allgemeinmedizin oder eine medizinische Fachkraft konsultiert?

◯ Nein
◯ Ja

Frage 1b_1: Wie viele Arzttermine hatten Sie in den letzten 3 Monaten bei Ihrem Hausarzt?

____

Frage 1b_2. Wie viele Termine hatten Sie in den letzten 3 Monaten bei Ihrer medizinischen Fachkraft?

____

Frage 2. Wie viele Termine hatten Sie in den letzten 3 Monaten mit einem Sozialarbeiter?

____

Frage 3. Wie viele Termine hatten Sie in den letzten 3 Monaten bei einem Physiotherapeuten? Oder mit einem Caesar-Therapeuten, Therapeuten Mensendieck oder einem Manualtherapeuten? Zählen Sie alle Termine bei diesen Therapeuten zusammen.

____

Frage 4. Wie viele Termine hatten Sie in den letzten 3 Monaten bei einem Ergotherapeuten?

____

Frage 5. Wie viele Termine hatten Sie in den letzten 3 Monaten bei einer Logopädin?

____

Frage 6. Wie viele Termine hatten Sie in den letzten 3 Monaten bei einer Ernährungsberaterin?

____

Frage 7. Wie viele Termine hatten Sie in den letzten 3 Monaten bei einem Homöopathen? Oder bei einem Akupunkteur? Zählen Sie alle Termine bei diesen Gesundheitsdienstleistern zusammen.

____

Frage 8. Wie viele Termine hatten Sie in den letzten 3 Monaten bei einem Psychologen? Oder bei einem Psychotherapeuten oder Psychiater? Zählen Sie alle Termine mit diesen Gesundheitsdienstleistern zusammen.

____

Frage 9 Wie viele Termine hatten Sie in den letzten 3 Monaten beim Betriebsarzt?

____

Frage 10a: Haben Sie in den letzten 3 Monaten häusliche Pflege erhalten?

Diese könnten sein:

- der Haushaltsführung, Haushaltshilfe, z.B. Staubsaugen, Bett machen, tägliche Einkäufe erledigen

- persänliche Pflege, z.B. Hilfe beim Baden oder Anziehen

- Krankenpflege z.B. Anlegen eines Verbandes, verabreichung von Medikamenten, Messen des Blutdrucks

◯ Nein
◯ Ja

Frage 10b. Welche Art von häuslicher Pflege haben Sie in den letzten 3 Monaten in Anspruch genommen?
Sie können mehr als 1 Kästchen ankreuzen.

◯ Haushaltsführung und Haushaltshilfe, Beispiel: Staubsaugen, Bett machen, tägliche Einkäufe erledigen
◯ Persönliche Pflege, Beispiel: Hilfe beim Baden oder Anziehen
◯ Krankenpflege, Beispiel: Anlegen eines Verbandes, Verabreichung von Medikamenten, Messen des Blutdrucks

Frage 10c_1. Wie viele Wochen haben Sie diese häusliche Hilfe in Anspruch genommen? Zählen Sie alle Wochen der letzten 3 Monate zusammen. Hinweis: Ein Zeitraum von 3 Monaten zählt 13 Wochen.

Antwort in: ___ Wochen in den letzten 3 Monaten

____

Frage 10c_2. Wie viele Wochen haben Sie diese persönliche Betreuung in Anspruch genommen? Zählen Sie alle Wochen der letzten 3 Monate zusammen. Hinweis: Ein Zeitraum von 3 Monaten zählt 13 Wochen.

Antwort in: ___ Wochen in den letzten 3 Monaten

____

Frage 10c_3. Wie viele Wochen haben Sie diese Krankenpflege in Anspruch genommen? Zählen Sie alle Wochen der letzten 3 Monate zusammen. Hinweis: Ein Zeitraum von 3 Monaten zählt 13 Wochen.

Antwort in: ___ Wochen in den letzten 3 Monaten

____

Frage 10d_1. Wie viele Stunden hauswirtschaftliche Hilfe haben Sie im Durchschnitt erhalten in diesen Wochen?

Antwort: im Durchschnitt ___ Stunden pro Woche

____

Frage 10d_2. Wie viele Stunden persönliche Betreuung haben Sie im Durchschnitt erhalten in diesen Wochen?

Antwort: im Durchschnitt ___ Stunden pro Woche

____

Frage 10d_3. Wie viele Stunden Krankenpflege haben Sie im Durchschnitt erhalten in diesen Wochen?

____

Medikamente

Frage 11a. Haben Sie in den letzten 3 Monaten Medikamente eingenommen?

Unter Medikamenten verstehen wir alle Medikamente, die Sie auf Rezept erhalten haben, sowie Medikamente, die Sie in der Apotheke oder Drogerie gekauft haben. Im Folgenden finden Sie drei Beispiele.

a) Wie lautet der Name des Medikaments? Sehen Sie sich die Verpackung an
z.B. Metoprolol (gegen Bluthochdruck), Furosemide (Diuretikum), Hydrokortison-Creme
b) Wie viel haben Sie jeweils eingenommen? Sehen Sie sich die Verpackung an
z.B. 100mg, 40mg, --
c) Wie viel haben Sie es pro Tag eingenommen? Schauen Sie auf die Verpackung
z.B. 1mal, 1mal, 1
d) An wie vielen Tagen in den letzten 3 Monaten haben Sie das Medikament eingenommen?
z.B. 90 Tage, 26 Tage (2x pro Woche, 13 Wochen), 14 Tage

◯ Nein
◯ Ja

Frage 11b. Welche Medikamente haben Sie in den letzten 3 Monaten eingenommen?
Achtung: Schauen Sie auf die Packung! Dort stehen die wichtigsten Informationen zur Medikamentenbeschreibung

Bitte geben Sie pro Medikament ALLE der folgenden Infos an:
- Name des Medikaments
- Firma des Medikaments
- Dosis
- Häufigkeit der Einnahme pro Tag
- Anzahl der Tage in den letzten 3 Monaten

Bitte schreiben Sie dies wie folgt:
z.B. Ibuprofen/ Sandoz Pharmaceuticals AG/ 400mg/ 1 pro Tag/ 5 Tage

Krankenhaus/-wagen

Frage 12a. Waren Sie in den letzten 3 Monaten im Krankenhaus oder der Notaufnahme (stationär, ambulant)?

◯ Nein
◯ Ja

Frage 12b. Wie oft haben Sie in den letzten 3 Monaten die Notaufnahme eines Krankenhauses aufgesucht?

____

Frage 13. Wie oft wurden Sie in den letzten 3 Monaten mit einem Krankenwagen ins Krankenhaus gebracht?

____

Frage 14a. Hatten Sie in den letzten 3 Monaten einen Termin in der Ambulanz des Krankenhauses? Hier geht es um Termine für Sie selbst bei einem Arzt. Zum Beispiel mit dem Kardiologen, Rheumatologen oder Neurologen.

◯ Nein
◯ Ja

Frage 14b. Welche Arten von Ärzten haben Sie in den letzten 3 Monaten im Krankenhaus aufgesucht? Und wie oft? Bitte geben Sie die Art von Arzt (z.B. Kardiologe) und die Zahl der Besuche (z.B. 1mal) an.

Frage 15a. Waren Sie in den letzten 3 Monaten zur tagesklinischen Behandlung im Krankenhaus? Sie sind also nicht über Nacht geblieben. Beispiele für tagesklinische Behandlungen sind Bluttransfusionen, Nierendialyse oder eine Chemotherapie.

◯ Nein
◯ Ja

Frage 15b. Um welche Art von Behandlung handelte es sich? Handelt es sich um mehr als eine Art von Behandlung? Geben Sie dann alle Arten von Behandlungen an.
z.B. Behandlung 1: xy, Behandlung 2: xy

Frage 15c. Wie oft mussten Sie in den letzten 3 Monaten für diese Behandlungen ins Krankenhaus?
z.B: 1mal für Behandlung 1, 2mal für Behandlung 2

Tagesklinische Behandlungen

Frage 16a. Waren Sie in den letzten 3 Monaten für eine tagesklinische Behandlung in einem anderen Krankenhaus? Sie sind also nicht über Nacht geblieben.

Sie waren zum Beispiel:

- in der Tagespflegeeinrichtung eines Wohn-/ Pflegeheims

- einer psychiatrischen Einrichtung.

- in der Tagespflegeeinrichtung eines Rehabilitationszentrums.

- einer anderen Einrichtung

◯ Nein
◯ Ja

Frage 16b. Um welche Art von Einrichtung handelte es sich? Kreuzen Sie die richtige Antwort an.
Sie können mehr als 1 Kästchen ankreuzen.

☐ Wohnheim oder Pflegeheim
☐ Rehabilitationszentrum
☐ Psychiatrische Einrichtung
☐ eine andere Einrichtung, bitte nachfolgend spezifizieren

Falls Sie eine andere Einrichtung für eine tagesklinische Behandlung handelt, spezifizieren Sie dies bitte nachfolgend

____

Frage 16c. Wie oft mussten Sie in den letzten 3 Monaten hierherkommen?
Haben Sie bei Frage 16b mehr als ein Kästchen angekreuzt? Dann geben Sie im Folgenden für jede Einrichtung an, wie oft Sie dort gewesen sind.

A) In einem Alten- oder Pflegeheim: ____ Mal in den letzten 3 Monaten

____

B) In einer Rehabilitationseinrichtung: ____ Mal in den letzten 3 Monaten

____

C) In einer psychiatrischen Anstalt: ____ Mal in den letzten 3 Monaten

____

D) In einer anderen Einrichtung (oben spezifiziert): ____ Mal in den letzten 3 Monaten

____

Frage 17a. Wurden Sie in den letzten 3 Monaten zur stationären Behandlung in ein Krankenhaus eingeliefert? Sie mussten also über Nacht bleiben. Zum Beispiel, weil Sie eine Operation hatten und nicht sofort nach Hause gehen konnten.

◯ Nein
◯ Ja

Frage 17b. Wie oft sind Sie in den letzten 3 Monaten zur stationären Behandlung ins Krankenhaus eingeliefert worden?

____ mal in den letzten 3 Monaten

____

Frage 17c. Wie lange haben Sie sich im Krankenhaus aufgehalten? Waren Sie in den letzten 3 Monaten mehr als einmal im Krankenhaus? Dann addieren Sie alle Tage zusammen.

___ Tage insgesamt in den letzten 3 Monaten

____

Frage 18a. Wurden Sie in den letzten 3 Monaten aus gesundheitlichen Gründen anderweitig eingewiesen? Zum Beispiel

- in einem Wohn-/Pflegezentrum
- einer psychiatrischen Einrichtung
- einem Rehabilitationszentrum
- einer anderen Einrichtung

◯ Nein
◯ Ja

Frage 18b. Um welche Art von Einrichtung handelte es sich? Sie können mehr als 1 Kästchen ankreuzen.

☐ Pflegezentrum oder Pflegeheim
☐ Rehabilitationszentrum
☐ Psychiatrische Einrichtung
☐ Eine andere Einrichtung

Falls Sie Frage 18b mit "eine andere Einrichtung" beantwortet haben, spezifizieren Sie dies bitte hier:

____

Frage 18c. Wie lange sind Sie schon in dieser Einrichtung? Haben Sie in Frage 18b mehr als ein Kästchen angekreuzt? Dann geben Sie bitte für jede Einrichtung an, wie lange Sie schon dort sind.

Waren Sie in den letzten 3 Monaten mehr als 1 Mal irgendwo? Zählen Sie dann alle Tage zusammen.

A) Im Pflegezentrum oder Pflegeheim

___ Tage in den letzten 3 Monaten

____

B) In der Rehabilitationseinrichtung:

___ Tage in den letzten 3 Monaten

____

C) In der psychiatrischen Anstalt:

___ Tage in den letzten 3 Monaten

____

D) In einer anderen Einrichtung:

___ Tage in den letzten 3 Monaten

____

Frage 19a. Haben Sie in den letzten 3 Monaten aufgrund Ihrer körperlichen oder psychischen Probleme Hilfe von einem Familienmitglied oder Bekannten erhalten?

Diese könnte wie folgt aussehen:

- Hauswirtschaftliche Hilfe - Beispiel: Staubsaugen, Bett machen, Einkaufen, Essen und Trinken zubereiten, Kinder betreuen
- Persönliche Pflege - Beispiel: Hilfe beim Duschen oder Anziehen, Hilfe beim Essen und Trinken oder beim Verabreichen von Medikamenten
- Praktische Hilfe - Beispiel: Unterstützung beim Spazierengehen, Ausflüge oder Besuche bei Bekannten, Besuche beim Hausarzt oder im Krankenhaus, Vermittlung von Hilfe oder Regelung finanzieller Angelegenheiten

◯ Nein
◯ Ja

Frage 19b. Welche Art von Hilfe haben Sie in den letzten 3 Monaten von Familienmitgliedern oder Bekannten erhalten? Sie können mehr als 1 Kästchen ankreuzen.

☐ Hauswirtschaftliche Hilfe - Beispiel: Staubsaugen, Bett machen, Einkaufen, Essen und Trinken zubereiten, Kinder betreuen
☐ Persönliche Pflege - Beispiel: Hilfe beim Duschen oder Anziehen, Hilfe beim Essen und Trinken oder beim Verabreichen von Medikamenten
☐ Praktische Hilfe - Beispiel: Unterstützung beim Spazierengehen, Ausflüge oder Besuche bei Bekannten, Besuche beim Hausarzt oder im Krankenhaus, Vermittlung von Hilfe oder Regelung finanzieller Angelegenheiten

Frage 19c. Wie viele Wochen haben Sie diese häusliche Pflege in Anspruch genommen? Zählen Sie alle Wochen der letzten 3 Monate zusammen. Hinweis: Ein Zeitraum von 3 Monaten zählt als 13 Wochen.

Hauswirtschaftliche Hilfe: ____ Wochen in den letzten 3 Monaten

____

Persönliche Betreuung: ____ Wochen in den letzten 3 Monaten

____

Praktische Hilfe: ___ Wochen in den letzten 3 Monaten

____

Frage 19d. Wie viele Stunden häusliche Pflege haben Sie im Durchschnitt erhalten in diesen Wochen?

Hilfe im Haushalt: im Durchschnitt ___ Stunden pro Woche

____

Persönliche Betreuung: im Durchschnitt ___Stunden pro Woche

____

Praktische Hilfe: im Durchschnitt ___ Stunden pro Woche

____

Frage 20a. Welches Verkehrsmittel haben Sie benutzt, um von zu Hause ins Krankenhaus zu gelangen?

◯ Nicht zutreffend
◯ Fußgänger
◯ Fahrrad
◯ Auto
◯ Öffentliche Verkehrsmittel
◯ Taxi
◯ Sonstige

Falls Sie bei Frage 20a mit "Sonstige" geantwortet haben, bitte hier spezifizieren:

____

Frage 20b. Wie groß war die einfache Entfernung zwischen Ihrem Wohnort und dem Krankenhaus?

Diese Entfernung betrug: ____Kilometer

____

Das war die letzte Frage.
Vielen Dank für das Ausfüllen des Fragebogens.
Wenn Sie Fragen oder Anmerkungen haben, können Sie diese hier auflisten.

Productivity Costs Questionnaire German_T0,2,3,4,5

Bitte füllen Sie die folgende Umfrage aus.Vielen Dank!

Productivity Costs Questionnaire

Bitte lesen Sie dies zuerst!

Worum geht es in diesem Fragebogen?
In diesem Fragebogen geht es um Ihre Gesundheit und Ihre Arbeit in den letzten drei Monaten.

Wie lange dauert das Beantworten der Fragen?
Das Beantworten der Fragen nimmt etwa 10 Minuten in Anspruch.

Gehen Sie einer bezahlten Beschäftigung nach?

◯ Nein
◯ Ja

Frage 1. Was ist Ihr Beruf?

____

Frage 2. Wie viele Stunden pro Woche arbeiten Sie? Zählen Sie nur die Stunden, für die Sie bezahlt werden.

____

Frage 3. Wie viele Tage pro Woche arbeiten Sie?

____

Frage 4. Sind Sie in den letzten 3 Monaten aufgrund einer Erkrankung nicht zur Arbeit gegangen?

◯ Nein
◯ Ja

Falls Sie Frage 4 mit Ja beantwortet haben:
Wie viele Tage haben Sie gefehlt?

Geben Sie nur die fehlenden Arbeitstage in den letzten 3 Monaten an.

____

Frage 5. Sind Sie vor den genannten 3 Monaten auf Grund einer Erkrankung nicht zur Arbeit gegangen? Diese Frage bezieht sich auf eine ununterbrochene Krankheitsphase.

◯ Nein
◯ Ja

Frage 6. Wann haben Sie sich krankgemeldet?

____

Frage 7. Gab es in den letzten 3 Monaten Tage, an denen Sie trotz körperlicher Beschwerden oder psychischer Probleme zur Arbeit gegangen sind?

◯ Nein
◯ Ja

Frage 8. Wie viele Arbeitstage waren Sie durch körperliche Beschwerden oder psychische Probleme beeinträchtigt? (Zählen Sie nur die Tage am Arbeitsplatz der letzten 3 Monaten.)

____

Frage 9. Fiel es Ihnen schwer, an den Tagen, an denen Sie durch diese Beschwerden und Probleme beeinträchtigt wurden, Ihr normales Arbeitspensum zu erledigen?
Wie viel Arbeit konnten Sie an diesen Tagen im Durchschnitt erledigen?

Sehen Sie sich untenstehende Zahlen an.
"10" bedeutet, dass Sie Ihr normales Arbeitspensum geschafft haben.
"5" bedeutet, dass Sie die Hälfte des normalen Arbeitspensums erledigt haben.
"0" bedeutet, dass an diesen Tagen überhaupt keine Arbeit erledigen konnten. Kreisen Sie die zutreffende Zahl ein.

0 5 10

(Place a mark on the scale above)

Erläuterung:

Selbst bei unbezahlter Arbeit können Sie durch körperliche Beschwerden oder psychische Probleme beeinträchtigt werden. Manchmal kann es daher vorkommen, dass man weniger schafft. Ihnen fällt es zum Beispiel schwer, Ihre Kinder zu betreuen oder ehrenamtlich tätig zu sein. Es kann auch sein, dass Ihnen der Einkauf oder die Gartenarbeit Mühe bereitet. Die folgenden Fragen beziehen sich auf diese Umstände.

Frage 10. Gab es Tage, an denen Sie gezwungen waren, Ihre unbezahlte Arbeit aufgrund körperlicher Beschwerden oder psychischer Probleme einzuschränken? Es kommt nur auf die Tage in den letzten drei Monaten an.

◯ Nein
◯ Ja

Frage 11. An wie vielen Tagen war dies der Fall? Zählen Sie nur die Tage in den letzten 3 Monaten.
_________ Tage

____

Frage 12. Stellen Sie sich vor, dass z. B. Ihr Partner, Ihre Partnerin, jemand aus Ihrer Familie oder ein Freund / eine Freundin Ihnen an diesen Tagen geholfen hat und dass er oder sie für Sie die unbezahlte Arbeit erledigt hat.

Wie viele Stunden haben diese Personen durchschnittlich für diese Arbeit aufgewendet?

Durchschnittlich _____ Stunden an diesen Tagen.

____

Das war die letzte Frage.
Vielen Dank!
Haben Sie Fragen oder Anregungen?
Möchten Sie vielleicht eine Frage stellen oder etwas kommentieren? Hier können Sie Ihre Fragen und/oder Kommentare vermerken:

Eq5d5l

Bitte füllen Sie den untenstehenden Fragebogen aus!Vielen Dank!

Die folgenden Fragen beziehen sich auf die Lebensqualität.
Bitte klicken Sie DAS Kästchen an, das Ihre Gesundheit HEUTE am besten beschreibt.

---

1) BEWEGLICHKEIT / MOBILITÄT

◯ Ich habe keine Probleme herumzugehen
◯ Ich habe leichte Probleme herumzugehen
◯ Ich habe mässige Probleme herumzugehen
◯ Ich habe grosse Probleme herumzugehen
◯ Ich bin nicht in der Lage herumzugehen

---

2) FÜR SICH SELBST SORGEN

◯ Ich habe keine Probleme, mich selbst zu waschen oder anzuziehen
◯ Ich habe leichte Probleme, mich selbst zu waschen oder anzuziehen
◯ Ich habe mässige Probleme, mich selbst zu waschen oder anzuziehen
◯ Ich habe grosse Probleme, mich selbst zu waschen oder anzuziehen
◯ Ich bin nicht in der Lage, mich selbst zu waschen oder anzuziehen

---

3) ALLGEMEINE TÄTIGKEITEN (z.B. Arbeit, Studium, Hausarbeit, Familien- oder Freizeitaktivitäten)

◯ Ich habe keine Probleme, meinen alltäglichen Tätigkeiten nachzugehen
◯ Ich habe leichte Probleme, meinen alltäglichen Tätigkeiten nachzugehen
◯ Ich habe mässige Probleme, meinen alltäglichen Tätigkeiten nachzugehen
◯ Ich habe grosse Probleme, meinen alltäglichen Tätigkeiten nachzugehen
◯ Ich bin nicht in der Lage, meinen alltäglichen Tätigkeiten nachzugehen

---

4) SCHMERZEN / KÖRPERLICHE BESCHWERDEN

◯ Ich habe keine Schmerzen oder Beschwerden
◯ Ich habe leichte Schmerzen oder Beschwerden
◯ Ich habe mässige Schmerzen oder Beschwerden
◯ Ich habe starke Schmerzen oder Beschwerden
◯ Ich habe extreme Schmerzen oder Beschwerden

---

5) ANGST / NIEDERGESCHLAGENHEIT

◯ Ich bin nicht ängstlich oder deprimiert
◯ Ich bin ein wenig ängstlich oder deprimiert
◯ Ich bin mässig ängstlich oder deprimiert
◯ Ich bin sehr ängstlich oder deprimiert
◯ Ich bin extrem ängstlich oder deprimiert

---

6) Wir wollen herausfinden, wie gut oder schlecht Ihre Gesundheit HEUTE ist.
Diese Skala ist mit Zahlen von 0 bis 100 versehen.

- 100 ist die beste Gesundheit, die Sie sich vorstellen können.
- 0 (Null) ist die schlechteste Gesundheit, die Sie sich vorstellen können. Bitte klicken Sie den Punkt auf der Skala an, der Ihre Gesundheit HEUTE am besten beschreibt.

0 - Schlechteste Gesundheit, die Sie sich vorstellen können	50	100 - Beste Gesundheit, die Sie sich vorstellen können

(Place a mark on the scale above)
