# Supplementary material for: Economic evaluation of a lifestyle intervention for individuals with overweight or obesity suffering from chronic low back pain (the BO2WL trial): a protocol for a health economic analysis
Source: BMJ Open. 2025 Jun 20;15(6):e098272. doi: 10.1136/bmjopen-2024-098272 (PMC12184353; doi:10.1136/bmjopen-2024-098272)
Supplement: Supplementary file 4 [file bmjopen-2024-098272.reviewer_comments.pdf]

PEER REVIEW HISTORY

BMJ Open publishes all reviews undertaken for accepted manuscripts. Reviewers are asked to complete a checklist review form and are provided with free text boxes to elaborate on their assessment. These free text comments are reproduced below.

ARTICLE DETAILS

Title (Provisional)

Protocol for the economic evaluation of a lifestyle intervention for individuals with overweight or obesity suffering from chronic low back pain (The BO2WL trial)

Authors

Schurz, Alexander Philipp; Deliens, Tom; Liechti, Melanie; Vanroose, Matteo; Clijsen, Ron; Nijs, Jo; Malfliet, Anneleen; Van Bogaert, Wouter; Clarys, Peter; Baur, Heiner; Taeymans, Jan; Lutz, Nathanael

---

VERSION 1 - REVIEW

Reviewer 1

Name Nguyen, Chi M.

Affiliation Indiana University Purdue University at Indianapolis, Biostatistics and Health Data Science

Date 20-Jan-2025

COI None

The authors clearly stated objectives of their study, explained details of their trial design, intervention, and planned economic approach and analysis.

However, their abstract needs editing to reflect their intervention more accurately in the "Methods and analysis". It'll be clearer to say that the trial includes 14 weeks of intervention and 52 weeks following the intervention; and the outcomes includes incremental CLBP-related care costs, QUALY and pain intensity.

There is just a few things that need more clarifying, as below.

On page 10, section "Study Interventions", more detail explanation on the intervention, i.e., the behavior weight reduction program, are desired. As it involves nutrition-specific inputs and increased physical activities, I wonder how the authors made sure that the intervened group of CLBP persons adhered to those. If the CLBP persons adhered to those, what would be the costs for their nutrition inputs, and for their extra time taking required physical activities? Will the costs be counted into intervention costs? It will be great if the authors make an additional list of all components of intervention costs.

In page 16, lines 177-179, the authors used a 3.5% discount rate for both costs and QUALY in CH as guided by NICE, but used 3.0% for costs and 1.5% for QUALY in BE guided by a BE-2012 guideline (as this study will evaluate the outcomes in 2026, I wonder if the BE guideline in 2012 is outdated). Please explain why you need to apply these rates in BE and can't apply the NICE's rate.

Reviewer **2**

Name **Turcu-Stiolica, Adina**

Affiliation **University of Medicine and Pharmacy of Craiova, Department of Biostatistics**

Date **03-Feb-2025**

COI **None**

Why do you choose Switzerland and Belgium for this study?

Please provide the reason of choosing different discount rates for CH and BE and why did you use these levels (3.5%, 3% and 1.5%).

The methods are described sufficiently to allow the study to be repeated, but the REDCap information must be provided in English.

RStudio will be used for the statistical analysis. What tests will be used to compare the two arms (intervention vs comparator)?

VERSION 1 - AUTHOR RESPONSE

Response to Reviewer 1

Dr. Chi M. Nguyen,

Thank you for your valuable feedback on our submitted health economic analysis plan. We have discussed all your points in our consortium and have added the point-by-point response below.

1. ***The authors clearly stated objectives of their study, explained details of their trial design, intervention, and planned economic approach and analysis. However, their abstract needs editing to reflect their intervention more accurately in the "Methods and analysis". It'll be clearer to say that the trial includes 14 weeks of intervention and 52 weeks following the intervention; and the outcomes includes incremental CLBP-related care costs, QUALY and pain intensity. There is just a few things that need more clarifying, as below.***

Thank you for this feedback. We have adapted the "Methods and analysis" section in the abstract accordingly (marked in yellow):

The trial consists of a 14-week intervention followed by a 52-week post-intervention follow-up. Primary outcomes include direct and indirect costs, as well as quality-adjusted life years (QALYs) and pain intensity. Cost data will be converted into 2026 Belgium euros (BE, CH) and 2026 Swiss Francs for CH, with a discount rate of 3.5% (CH, for both costs and effects) and 3 and 1.5% (BE, for costs and effects respectively). Incremental cost-utility and cost-effectiveness ratios will be calculated from a societal perspective for each country.

2. ***On page 10, section "Study Interventions", more detail explanation on the intervention, i.e., the behavior weight reduction program, are desired. As it involves nutrition-specific inputs and increased physical activities, I wonder how the authors made sure that the intervened group of CLBP persons adhered to those. If the CLBP persons adhered to those, what would be the costs for their nutrition inputs, and for their extra time taking required physical activities? Will the costs be counted into intervention costs? It will be great if the authors make an additional list of all components of intervention costs.***

We acknowledge the need for further details regarding the behavioural weight reduction program, particularly in relation to adherence and cost considerations.

Adherence to the intervention is monitored by the treating physiotherapists, who assess compliance with the prescribed treatments throughout the intervention period. However, we have decided not to evaluate the costs associated with nutritional intake and physical activity for the following reasons:

- **Participant burden**: Participants are already required to complete a substantial number of questionnaires at multiple assessment time points and throughout the treatment. To minimize additional burden, food diaries are only evaluated for three days before and at the end of the intervention. Consequently, detailed assessments of nutritional costs and costs related to increased physical activity are not included.
- **Risk of double counting**: Assessing the costs of nutrition and physical activity may introduce methodological challenges. Participants with reduced pain levels might naturally engage in more physical activity, potentially increasing their associated costs. Including these costs in the analysis could distort the cost-effectiveness evaluation by conflating treatment effects with lifestyle changes.

- **Focus on intervention costs**: The direct costs of physiotherapy sessions are covered by the study. Given that we anticipate a significant economic burden from productivity losses, our cost analysis prioritizes this aspect rather than the costs of individual lifestyle modifications.
  We acknowledge the importance of a comprehensive cost analysis and have added a column in Table 2 to outline the components of intervention costs, thereby enhancing transparency. Additionally, we have added a section on the evaluation of therapy adherence (marked in yellow).

**Cost category**	**Category**	**Switzerland**	**Belgium**
**Intervention costs**	Treatment	17 Physiotherapy sessions: Monetised according to current physiotherapy tariffs (40, 41, 42)<br>1 Home-Based Session: Not monetised, as the physiotherapist is not involved.<br>Additional Costs for Lifestyle Interventions: Not collected	

We have also adapted the section on *Study intervention* by adapting it as depicted below:

Participants are blinded and randomly assigned to either the intervention group or the control group, with study assessors and statistician blinded to group allocation. All participants receive the same 14-week treatment depending on their group allocation. Participants of the intervention group will receive up to 17 individually based treatment sessions, supervised by experienced physiotherapists who have been trained a priori by the research team for these treatments, along with one home-based session. Treatment sessions will include PNE, CTET and a behavioural weight reduction program. The latter is integrated alongside CTET therapy sessions with various nutrition-specific inputs and actions to increase physical activity. The participants of the control group also receive 17 individually based treatment sessions and one home-based session consisting of PNE and CTET therapy alone. Physical therapists will document the content of each treatment session as required by their workplace. After treatment completion, the physical therapist will be asked to complete a questionnaire on therapy adherence and compliance of the participant

(number of sessions, sessions per intervention, total duration, duration per intervention, overall compliance to the intervention (control or intervention group).

3. ***In page 16, lines 177-179, the authors used a 3.5% discount rate for both costs and QUALY in CH as guided by NICE, but used 3.0% for costs and 1.5% for QUALY in BE guided by a BE-2012 guideline (as this study will evaluate the outcomes in 2026, I wonder if the BE guideline in 2012 is outdated). Please explain why you need to apply these rates in BE and can't apply the NICE's rate.***

Thank you for your feedback regarding the discount rates. The Belgium guideline from 2012 is at present still the most recent one. However, to improve the comparability of the results despite the differences in healthcare systems, we have adapted the discount rates as described below:

- We will use the discount rates of 3.5% for both countries in the base case for costs and effects, as recommended in the NICE guidelines.
- As already described in the manuscript, we plan to conduct scenario analyses with varying discount rates. CH and BE have different health systems and economic realities which can affect the relevance of a specific discount rate. To improve the accuracy of the analysis, we will further conduct one scenario analysis using the Belgium discount rates of 3% for costs and 1.5% for effects for the Belgium data.

Therefore, we have implemented this in the following sections of the manuscript (marked in yellow):

Abstract:

Cost data will be converted into 2026 Belgium euros (BE, CH) and 2026 Swiss Francs for CH, with a discount rate of 3.5%. Incremental cost-utility and cost-effectiveness ratios will be calculated from a societal perspective for each country.

Table 1:

Discount rate	CH/BE: 3.5% costs and effects
Sensitivity analyses	Probabilistic: Variation of all input variables in nonparametric bootstrapping<br>Deterministic: One-way sensitivity analysis<br>Scenario analysis: Variation of discount rate (e.g. country specific rates BE: 3.0% costs, 1.5% effects)

Economic data analysis

"Discounting of costs and outcomes over a time horizon exceeding twelve months will follow NICE guidelines, with a discount rate of 3.5% applied to both costs and outcomes in CH and BE. Country-specific discount rates for BE will be applied in the scenario analysis using 3.0% for costs and 1.5% for outcomes."

And at the end of the economic data analysis section:

„In the scenario analysis, varying discount rates (e.g. country-specific discount rates for BE with 3.0% for costs and 1.5% for outcomes) and different value sets for the EQ-5D-5L questionnaire will be applied to assess their effect on the outcome of cost-effectiveness."

Response to Reviewer 2

Dr. Adina Turcu-Stiolica,

We deeply thank you for the important feedbacks regarding our health economic analysis plan. We have addressed each of your feedback items below:

1. ***Why do you choose Switzerland and Belgium for this study?***

   The research team comprises an international consortium of experts from Belgium and Switzerland, specializing in pain management (Belgium), lifestyle interventions (Belgium, and health economics (Switzerland). To generate evidence that is more generalisable across different populations and countries, we opted for a multinational study design and chose to conduct the trial in these two countries. This approach enhances the robustness of the trial's conclusions and ensures a broader applicability of the findings.

2. ***Please provide the reason of choosing different discount rates for CH and BE and why did you use these levels (3.5%, 3% and 1.5%).***

   Thank you for your feedback regarding the discount rates. This issue was also raised by the other reviewer. The most recent Belgian guideline available remains the 2012 version. However, to enhance the comparability of results despite variations in healthcare systems, we have adjusted the discount rates as follows:

   - In the base case, we will apply a uniform discount rate of 3.5% for both costs and effects in both countries, following the recommendations of the NICE guidelines.
   - As outlined in the manuscript, we will also perform scenario analyses using alternative discount rates. Given the economic differences between CH and BE,

certain discount rates may be more relevant in one context than the other. To ensure a more precise analysis, we will include an additional scenario analysis that applies BE specific discount rates - 3% for costs and 1.5% for effects - exclusively to the BE data.

These adjustments have been incorporated into the manuscript and are highlighted accordingly.

Abstract:

Cost data will be converted into 2026 Belgium euros (BE, CH) and 2026 Swiss Francs for CH, with a discount rate of 3.5%. Incremental cost-utility and cost-effectiveness ratios will be calculated from a societal perspective for each country.

Table 1:

Discount rate	CH/BE: 3.5% costs and effects
Sensitivity analyses	Probabilistic: Variation of all input variables in nonparametric bootstrapping<br>Deterministic: One-way sensitivity analysis<br>Scenario analysis: Variation of discount rate (e.g. country specific rates BE: 3.0% costs, 1.5% effects)

Economic data analysis

"Discounting of costs and outcomes over a time horizon exceeding twelve months will follow NICE guidelines, with a discount rate of 3.5% applied to both costs and outcomes in CH and BE. Country-specific discount rates for BE will be applied in the scenario analysis using 3.0% for costs and 1.5% for outcomes."

And at the end of the economic data analysis section:

„In the scenario analysis, varying discount rates (e.g. country-specific discount rates for BE with 3.0% for costs and 1.5% for outcomes) and different value sets for the EQ-5D-5L questionnaire will be applied to assess their effect on the outcome of cost-effectiveness."

3. ***The methods are described sufficiently to allow the study to be repeated, but the REDCap information must be provided in English.***

Thank you for your feedback on the English versions of the questionnaires. However, we have included freely accessible sample versions of the EQ-5D-5L, iMCQ, and iPCQ questionnaires in the supplementary materials. These samples, provided by the respective development companies, are not intended for data collection but serve to enhance understanding of the collected data.

4. ***RStudio will be used for the statistical analysis. What tests will be used to compare the two arms (intervention vs comparator)?***

Thank you for your feedback.

The following sections were included already to describe the planned statistical analysis. We believe that the current descriptions provide sufficient detail, although variations may arise depending on the nature of the data collected in the trial. We hope this level of detail meets your expectations and that you find it satisfactory.

- **Section "Economic data collection and management":**

  "RStudio version 2024.04.1+748 or higher will be used for the statistical analysis (27)."

  We kept the section at the beginning of this paragraph as recommended in the reporting guidelines.

- **Section "Economic data analysis":**

„The comparison of mean costs between the groups will be initially conducted using Ordinary Least Squares (90) regression, adjusting for the minimisation variables used in the randomisation procedure. Subsequently, the residuals from the Ordinary Least-Squares (OLS) regression model will be examined to assess their distribution. Based on this assessment, it will be determined whether OLS is appropriate or if an alternative regression model, such as Generalised Linear Models, should be considered. To analyse the outcome variables, an appropriate regression model will also be employed to account for any imbalances in baseline utility and the minimisation variables from the randomisation process (91)."

"A nonparametric bootstrapping method will be employed to assess the sampling uncertainty associated with the mean ICER and ICUR values. This procedure will generate 10,000 estimates of incremental costs and effects (98) and graphically presented in a cost-effectiveness acceptability curve (CEAC).

Subgroup analyses will be performed using the complete cases to explore variations in cost-effectiveness and cost-utility across Switzerland and Belgium and distinct patient

subgroups (e.g. different weight categories). In the scenario analysis, varying discount rates and different value sets for the EQ-5D-5L questionnaire will be applied to assess their effect on the outcome of cost-effectiveness.“
